# Skin Regeneration in Adult Axolotls: A Blueprint for Scar-Free Healing in Vertebrates

**DOI:** 10.1371/journal.pone.0032875

**Published:** 2012-04-02

**Authors:** Ashley W. Seifert, James R. Monaghan, S. Randal Voss, Malcolm Maden

**Affiliations:** 1 Department of Biology, University of Florida, Gainesville, Florida, United States of America; 2 Department of Biology, University of Kentucky, Lexington, Kentucky, United States of America; 3 Spinal Cord and Brain Injury Research Center, University of Kentucky, Lexington, Kentucky, United States of America; The University of Hong Kong, Hong Kong

## Abstract

While considerable progress has been made towards understanding the complex processes and pathways that regulate human wound healing, regenerative medicine has been unable to develop therapies that coax the natural wound environment to heal scar-free. The inability to induce perfect skin regeneration stems partly from our limited understanding of how scar-free healing occurs in a natural setting. Here we have investigated the wound repair process in adult axolotls and demonstrate that they are capable of perfectly repairing full thickness excisional wounds made on the flank. In the context of mammalian wound repair, our findings reveal a substantial reduction in hemostasis, reduced neutrophil infiltration and a relatively long delay in production of new extracellular matrix (ECM) during scar-free healing. Additionally, we test the hypothesis that metamorphosis leads to scarring and instead show that terrestrial axolotls also heal scar-free, albeit at a slower rate. Analysis of newly forming dermal ECM suggests that low levels of fibronectin and high levels of tenascin-C promote regeneration in lieu of scarring. Lastly, a genetic analysis during wound healing comparing epidermis between aquatic and terrestrial axolotls suggests that matrix metalloproteinases may regulate the fibrotic response. Our findings outline a blueprint to understand the cellular and molecular mechanisms coordinating scar-free healing that will be useful towards elucidating new regenerative therapies targeting fibrosis and wound repair.

## Introduction

Among its many functions, the skin is primarily responsible for maintaining the structural and physiological barrier between an organism’s internal and external environment. As the first line of defense against external insult the skin is injured more frequently than any other tissue and resulting damage, while repairable, leads to permanent scarring in mammals [1]. At least 100 million people in the developed world acquire scars each year in response to trauma and surgery and the result is a spectrum of pathologies from thin line surgical scars to hypertrophic and chronic non-healing wounds [2], [3], [4]. Adding to this medical burden, burn injuries, which often elicit an over-exuberant fibrotic response, result in hypertrophic scarring with treatment costs in the US alone accounting for $4 billion annually [5]. While not as complex as regenerating a human digit or limb, the ability to develop regenerative strategies that lead to scar-free healing in adult skin remain tantalizingly out of reach. Understanding how to coax the natural wound repair process towards a regenerative outcome remains the grail of wound healing research.

Our knowledge of the molecular and cellular events during mammalian tissue repair is extensive (see refs [1], [6], [7], [8]) and yet, even with such broad understanding of the wound repair process, regenerative medicine has failed to develop therapies that can perfectly regenerate skin. This stems partly from the dynamic reciprocity of cellular interactions and signaling pathways and partly from a lack of appropriate models to observe these interactions in a regenerative environment [9]. While wound repair in fetal mammals [10], [11], [12], [13], [14] and marsupials [15] has provided insight into the cellular and molecular regulation of scar-free healing, comparisons of wound repair between fetal mammals and adults has limitations, both biological and practical [16]. The developing fetus, at the time when it heals scar-free, has an immature endocrine system, is immuno-incompetent, is contained in a moist sterile environment, and its cells are in a state of chronic hypoxia [16]. Adult skin is more completely differentiated and adult wounds are open to desiccation and infection, two factors that seriously complicate wound repair. Other promising models of scar-free healing, such as the MRL mouse, which share the ability to regenerate ear punches with rabbits, hares, pikas, cows, pigs and cats [17], [18], [19] has proven less than perfect when challenged to heal excisional skin wounds [20], [21] casting doubt on the special regenerative powers of this inbred mouse model.

Compared to other vertebrates, urodeles possess the amazing capacity to regenerate their limbs, hearts, lenses, spinal cords, tails, internal organs and joints. Observations from studies examining limb regeneration have been extrapolated to the skin, but direct comparisons to the processes of cutaneous wound repair have rarely been made [22]. Studies examining limb wounds have yielded insight into the process of re-epithelialization [23], [24], [25], [26] and regeneration of the basement membrane [27], [28], but the dynamics of dermal regeneration have remained obscure. Direct study of wound repair in urodele skin outside of regeneration fields like the limb and tail, however, has not been undertaken.

Given their seemingly absolute powers of regeneration, a recurring question has been whether wounds made outside of regenerating structures (e.g. limbs and tails) in adult urodeles are capable of scar-free healing or, like adult anurans, heal with a scar [29]. In this study we examined full thickness excisional (FTE) wound healing of dorsal back skin in adult axolotls. Using an established mammalian excisional wound model to directly characterize cutaneous wound healing in adult axolotls, we examined hemostasis, inflammation, new tissue formation and remodeling processes. Additionally, we induced metamorphosis in adult axolotls to test the hypothesis that loss of larval skin characters and transition to a terrestrial form results in fibrotic scarring following FTE flank wounds. Here we demonstrate that both aquatic and terrestrial axolotls are capable of perfect, scar-free skin regeneration. We discuss these findings in the context of mammalian wound repair and present a blueprint for investigating the cellular and molecular mechanisms that regulate scar-free skin healing in adult vertebrates.

## Results

### Full Thickness Excisional Flank Wounds are Perfectly Regenerated in Adult Axolotls

**Figure 1:**
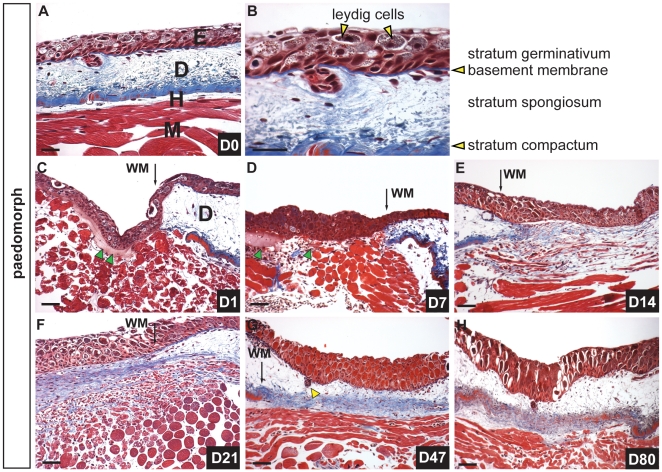
Scar-free healing of full thickness excisional wounds in adult axolotls. A) Masson’s trichrome staining of uninjured dorsal flank skin in axolotls showing epidermis (E), dermis (D), hypodermis (H) and underlying muscle (M). B) Magnified image of epidermis and dermis. The epidermis is pseudo-stratified and contains epithelial cells and leydig cells (yellow arrows) while the dermis is divided into the stratum spongiosum (containing glands and dermal fibroblasts) atop the densely compacted ECM of the stratum compactum. C-H) Scar-free healing over 80 day period following full thickness excisional wounding. C) One day post injury (dpi) the wound bed is completely re-epithelialized. Some blood plasma has accumulated beneath the neoepidermis (green arrows) (wound margin = WM). D) Seven dpi there is little evidence of a fibrin clot between the epidermis and underlying muscle and no new ECM has been deposited. Green arrows depict residual blood plasma. E) Fourteen dpi fibroblasts are visible beneath the epidermis where new ECM is deposited (blue staining) and muscle cells are fragmenting into individual myoblasts. F) Twenty-one dpi a thick band of transitional matrix is visible beneath the epidermis. Collagen is visible within the regenerating muscle. G) Forty-seven dpi the underlying muscle has completely regenerated and is devoid of collagen. Skin glands (yellow arrow) have regenerated and descended from the epidermis. The dermal stratum spongiosum has reformed and the stratum compactum is coalescing. H) Eighty dpi all skin layers have regenerated. Scale bars = 100 µm.

Adult axolotls (*Ambystoma mexicanum*) are aquatic and paedomorphic, exhibiting several juvenile features as adults (e.g. retention of leydig cells, pseudo-stratified epidermis, external gills). Although the dermis of adult axolotl skin is typical for amphibians, the epidermis is pseudo-stratified and lacks a well-defined stratum corneum (Figure 1A, B). Above the stratum germinativum, epithelial cells are interspersed with leydig cells (specialized cells containing highly granulated cytoplasm) that are characteristic of larval amphibian skin (Figure 1B and [30], [31]). The dermis contains epidermally-derived mucous and granular glands that are embedded within the stratum spongiosum, a loose network of thin collagen fibers and fibroblasts that lies above the stratum compactum (Figure 1A, B and Figure S1A, B). The stratum compactum forms a thickened sheet of compressed collagen fibers that sits atop hypodermis and separates the skin from the underlying muscle (Figure 1A).

**Figure 2:**
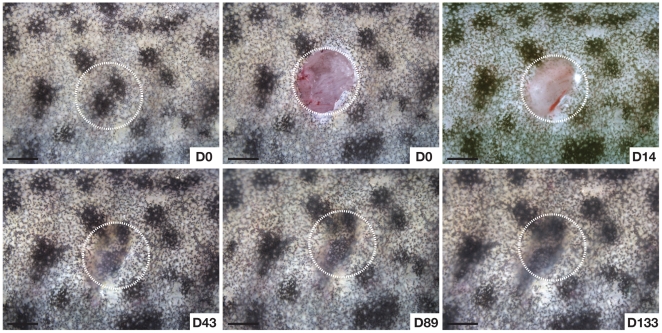
Scar-free healing in adult axolotls. Regeneration of dorsal flank skin following 4 mm full thickness biopsy punch wounding. White circles depict area of original injury. Individual pigment cells are visible at D14 in the overlying epithelium. Regeneration and scar-free healing at D89. Contraction is evident at the wound edges after D14. Scale bars = 2.0 mm.

It has been reported that wounds made outside of regeneration fields (i.e. limbs, tail, head) heal with a scar [32] and to date, this controversy remains unresolved. To directly test the hypothesis that axolotls can perfectly regenerate (i.e. heal scar-free) full thickness excisional (FTE) wounds, we made circular 4mm FTE wounds through the dermis into the dorsal flank muscle (n = 12) and observed the wound healing process over 180 days (Figure 1C-H, Figure 2, Figures S2). In response to injury blood flowed into the wound bed, clotted, but did not form a scab (Figure 2). Epithelial cells from the wound margins migrated across the underlying muscle and accumulated plasma and completely re-epithelialized the wound within 24hrs (Figure 1C, green arrows indicate plasma). Following re-epithelialization numerous blood cells (leukocytes and erythrocytes) were apparent in the wound bed and the neoepidermis remained in close proximity with the underlying muscle and plasma over the next 7–10 days (Figure 1D). Fourteen days post injury we observed dermal fibroblasts in the wound bed and Masson’s Trichrome staining revealed newly deposited extracellular matrix (ECM) (Figure 1E). Twenty-one days post injury muscle fibers continued to fragment liberating mono-nucleate cells into the surrounding tissue and a robust ECM formed between the epidermis and muscle (Figure 1E, F). Forty-seven days post-injury new epidermal organs were present in the regenerated stratum spongiosum (Figure 1G and Figure S2A). The stratum compactum, however, had not completely regenerated and this was clearly evident at its margins (Figure 1G and Figure S2B). Although the timing to regeneration varied among individuals, full thickness skin, including epidermal organs and underlying muscle, was completely regenerated by 80 days (Figure 1H and Figure 2). Maturation and development of glands and the stratum compactum continued over the next 100 days (Figure S2C).

### New ECM Deposition Corresponds with Formation of the Lamina Densa

**Figure 3:**
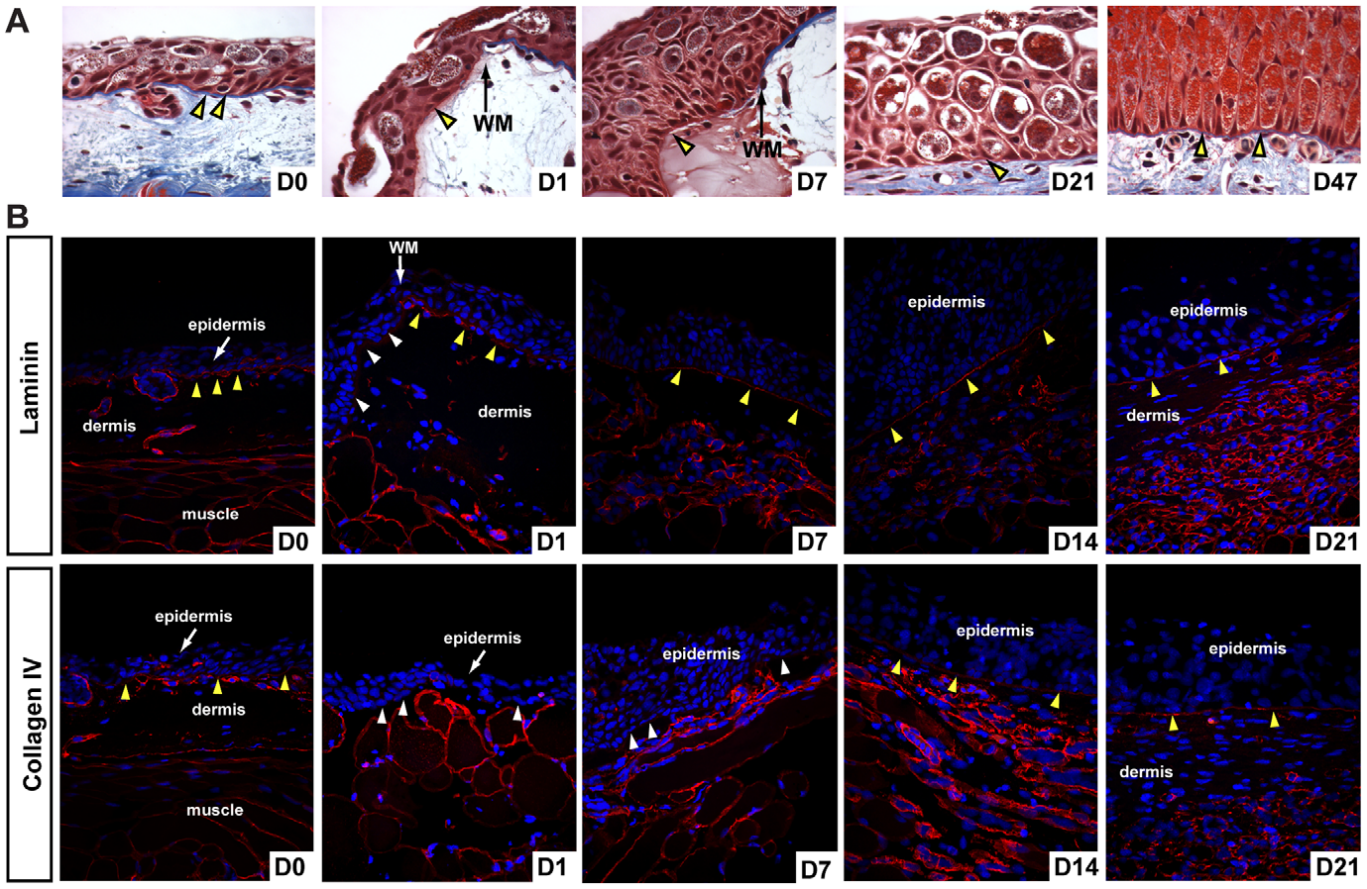
Lamina lucida and lamina densa regenerate before new ECM deposition. A) Histological examination of basement membrane (BM) regeneration in axolotls. The uninjured BM is visible as a thick blue-stained fibrous band (yellow arrows). An immature BM has begun to reform (yellow arrow D1) after re-epithelialization and is visible at the wound margin (WM) in contrast to the uninjured BM. The regenerated BM is visible at D47. Yellow arrows at D7 and D21 indicate reforming BM. B) Examination of lamina lucida (laminin) and lamina densa (collagen type IV) during basement membrane regeneration. The uninjured BM is positive for laminin and collagen type IV (yellow arrows) as are the basement membranes surrounding glands and muscle fibers. Following re-epithelialization the basal lamina of the epidermis is negative for laminin and collagen type IV (white arrows) and this is clearly evident at the wound margin (WM). Seven days post injury the BM stains strongly for laminin indicating reformation of the lamina lucida, while staining for collagen type IV is punctuated. The lamina densa is regenerated by D14 based on continuous collagen type IV staining and persists during dermal regeneration.

Previous work in regenerating newt limbs suggested that reformation of the basement membrane (BM) facilitates dermal regeneration and its delayed formation permits blastema formation [28]. We followed BM regeneration after re-epithelialization and asked whether it occurred prior to the onset of dermal regeneration in excisional flank wounds. In uninjured skin the BM is visible as a thick fibrous band separating epidermis from dermis and is continuous except where mucous glands interject into the epidermis (Figure 3A). Following re-epithelialization histological staining revealed a thin, immature structure beneath the new epidermis (Figure 3A). The BM continued to mature and was completely regenerated at least 47 days after wounding (Figure 3A; yellow arrows D47). Interestingly, complete regeneration of the BM corresponded to regeneration of the dermis (except for stratum spongiosum) (Figure 3A and Figure 1G).

In order to test if assembly and maturation of the BM corresponded to the onset of ECM deposition we analyzed formation of the lamina lucida and lamina densa using antibodies that recognize laminin (lamina lucida) and collagen type IV (lamina densa) (Figure 3B). Both proteins were detectable in the BM of uninjured skin, and surrounding glands and muscle fibers (Figure 3B). Following re-epithelialization, basal epidermal cells were negative for both laminin and collagen IV (Figure 3B; white arrows). We detected strong and continuous laminin staining beneath the epidermis 7 days post injury indicating lamina lucida reformation (Figure 3B). Collagen IV was not detected continuously beneath the epidermis until D14 (corresponding to the onset of ECM deposition) (Figure 3B).

In addition to protein localization we tracked epidermal gene expression of the BM components *collagen type IV* and *laminin alpha* 1 (*Lama1*), *laminin beta* 1 (*Lamb1*), *laminin gamma* 1 (*Lamc1*), which together form the protein laminin*-*111 (Table S1). Expression of all three laminin-111 subunits was unchanged during re-epithelialization (Table S1). Expression of both *alpha-1* and *beta-1* components of laminin-111 increased 5.6-fold and 4.1-fold respectively between D1 and D3, and continued increasing at D7 post injury (Table S1). In the skin basement membrane of humans and mice, two hetero-trimeric collagen type IV molecules consisting of alpha1(IV)_2_/alpha2(IV) and alpha5(IV)_2_/alpha6(IV) chains exist [33]. We examined expression of each of these alpha chains following injury and found that expression of *alpha1* and *alpha2* chains were down regulated following injury, remained below baseline levels until D3, after which they returned to baseline levels at D7 post injury (Table S1). Whereas expression of the *alpha6* chain remained unchanged, the *alpha5* chain was upregulated 4.9-fold at D1 and sustained through D7 (Table S1). Taken together, these results demonstrate that regeneration of the BM proceeds through formation of the lamina lucida followed by production of the lamina densa and that complete lamina densa formation corresponds to the onset of ECM production in the wound bed.

### Metamorphic Axolotls Exhibit a Delay in Skin Regeneration Compared to Paedomorphic Axolotls

**Figure 4:**
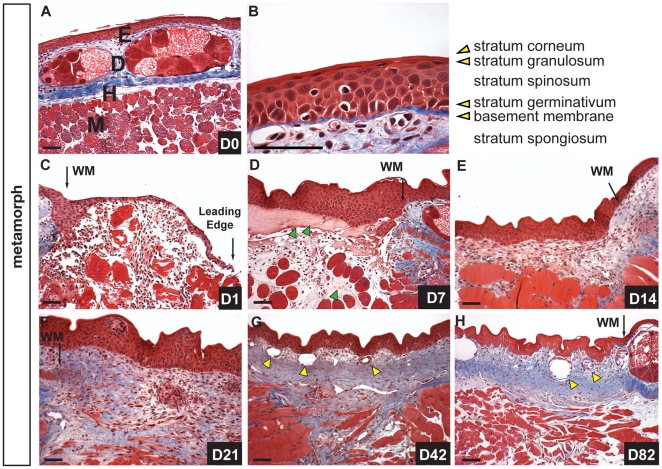
Metamorphic axolotl skin heals scar-free but slower compared to paedomorphs. A–B) Morphology of uninjured axolotl skin after metamorphosis. A) Masson’s trichrome staining showing epidermis (E), dermis (D) containing enlarged granular glands in stratum spongiosum atop stratum compactum, hypodermis (H) and muscle (M). B) High magnification of epidermis showing stratified epithelium. Leydig cells have disappeared and the epidermis now contains a well-defined stratum spinosum, granulosum and corneum. C-H) Wound healing following FTE wounds over 82-day period. C) One day post injury (dpi) epithelial cells have begun to migrate but the wound is not re-epithelialized. Erythrocytes are visible in the wound bed and between muscle fibers undergoing histolysis. D) Re-epithelialization is complete 3 dpi and at 7 dpi coagulated plasma (green arrows), erythrocytes and leukocytes are visible in the wound bed. E) Fourteen dpi fibroblasts are visible in the wound bed and new ECM is deposited (blue staining). The wound margin (WM) is still clearly visible. F) The wound bed 21 dpi is rich in ECM. This ECM extends deep into the underlying muscle fibers which are fragmenting into myoblasts. G). Regenerating glands (yellow arrows) are present in the dermis 42 dpi and the stratum spongiosum is beginning to develop. The underlying muscle continues to remain damaged with deep pockets of collagen persisting beneath the wound. H) Eighty dpi the dermis is partially regenerated but the stratum compactum has not coalesced. Some collagen still persists deep in the muscle and both mucous and granular glands have regenerated (yellow arrows). Scale bars = 100 µm.

While adult mammals are incapable of regenerating full thickness skin wounds, fetal mammals exhibit scarless healing of similar type wounds [12]. Similarly, while pre-metamorphic anurans heal scar-free, post-metamorphic anurans have been documented to heal flank wounds through scar formation [29]. Adult axolotls retain several larval skin features (e.g. leydig cells, pseudo-stratified epithelium), thus we asked if these characteristics facilitate their ability to heal wounds scar-free. To test this hypothesis we exploited the fact that normally aquatic axolotls retain the ability to undergo metamorphosis to a terrestrial form through administration of thyroxine and we induced metamorphosis in adult axolotls (controlling for age and size with sibling paedomorphs). Comparing uninjured epidermis between both forms we noted two major differences; first, granular glands that occupied relatively little space in the paedomorph dermis were greatly enlarged and occupied most of the stratum spongiosum while mucous glands appeared similar in form between morphs (Figure 4A and Figure S3A). Second, the epidermis no longer contained leydig cells and had transitioned to a completely stratified epithelium exhibiting a well-defined stratum germinativum, stratum spinosum, stratum granulosum and stratum corneum (Figure 4B).

We next examined wound repair in metamorphs following 4mm full thickness excisional wounds over 180 days (Figure 4C-H and Figure S3B-E). Twenty-four hours post injury re-epithelialization was not complete and the leading edge of migrating epidermal cells was visible both macroscopically and in section (Figure 4C). Re-epithelialization was complete by 72hours post injury and the epidermis had re-stratified to establish apical/basal polarity along the regenerating basement membrane (Figure 4D). Seven days post injury the wound bed contained plasma beneath the epidermis and within the fragmented muscle, along with large numbers of erythrocytes and leukocytes (Figure 4D). ECM deposition began as it had in the paedomorph approximately 10–14 days post injury and we noted that it appeared to extend deep into the muscle (Figure 4E). Twenty-one days post injury the ECM was dense within the wound bed, new vasculature was present in both the epidermis and ECM, muscle fibers continued to fragment and aggregations of cells appeared in the epidermis suggesting glands were beginning to regenerate (Figure 4F and Figure S3B).

Complete dermal regeneration was delayed in metamorphs (compare Figure 1G and Figure 4G). While epidermal organs regenerated in both forms after 40 days, the wound bed and underlying muscle still contained densely compacted extracellular matrix in metamorphs (Figure 4G and Figure S3C). After 80+ days the stratum spongiosum had regenerated but the stratum compactum remained incomplete (Figure 4H). After 120 days, the wound site resembled an 80-day regenerating wound in paedomorphs and a few collagen deposits still persisted in the underlying muscle (Figure S3D). Fibrosis was not resolved until at least 148 days and while mucous glands regenerated to pre-wound size, granular glands remained small even after 148 days (Figure S3E). Taken together these findings suggest that flank skin in adult metamorphic axolotls can completely regenerate following FTE wounding, but the time required to regenerate both the stratum compactum and mature granular glands is lengthened compared to paedomorphs.

### The Rate of Re-epithelialization is Slower in Metamorphs

**Figure 5:**
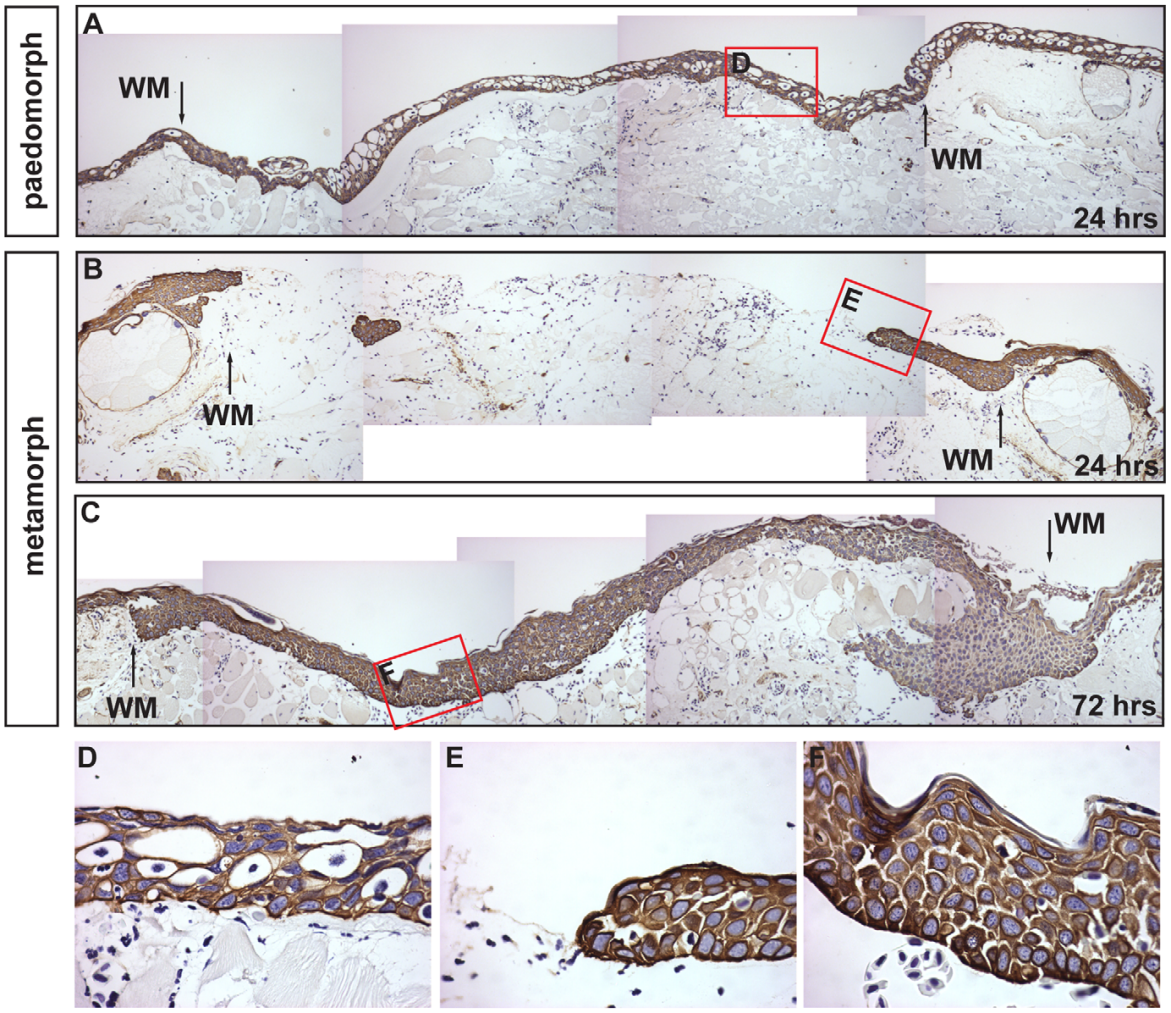
The rate of re-epithelialization is slower in metamorphs compared to paedomorphs. Epidermis was labeled with a pan-cytokeratin antibody. A) The wound bed is completely re-epithelialized 24 hrs after injury in paedomorphs. The wound margins (WM) are visible where the stratum compactum is disrupted. B, C) Wound edge keratinocytes have just begun migrating 24 hrs dpi and re-epithelialization is complete by 72 hrs dpi in metamorphs. D) Leydig cells are present in the paedomorph neoepidermis. E) The leading edge of migrating metamorph keratinocytes. The epidermal cells appear to move as a group of cells with one cell at the leading edge. F) After re-epithelialization is complete in metamorphs the epidermis becomes re-stratified.

Following injury, re-epithelialization of the wound bed is necessary to re-establish epidermal integrity. In various rodent species, untreated FTE wounds re-epithelialize between 3 and 7 days (personal obser.). In contrast, aquatic axolotls re-epithelialize 4mm flank wounds in ∼18hrs (Figure 1C and Figure S4). We compared the rate of re-epithelialization between paedomorphs and metamorphs to test whether the completely stratified epidermis of terrestrial axolotls affected its ability to cover the wound bed. Using an antibody to a wide-spectrum of cytokeratins we labeled the epidermis 24 and 72hrs after injury (Figure 5). Compared to the completely re-epithelialized wound bed of paedomorphs, wound edge epithelial cells in metamorphs had just begun migrating 24 hrs after injury (Figure 5A, B). Metamorph re-epithelialization was complete by 72 hrs (Figure 5C). Examination of the wound epidermis in paedomorphs showed leydig cells present in the new epidermis (Figure 5D). The leading edge of metamorph epidermis appeared pseudo-stratified (reminiscent of paedomorph epidermis without leydig cells) and moved as a sheet of cells with a single cell at the leading edge (Figure 5E). Following re-epithelialization, the wound epidermis re-established a stratified epithelial morphology (Figure 5F). In conjunction with this delay in re-epithelialization we observed a concomitant delay in appearance of the BM components laminin and collagen type IV reflecting the dependency of BM reassembly on the timing of complete re-epithelialization (data not shown). We examined the expression of the same molecular components of laminin and collagen type IV as we did for paedomorphs and did not detect a significant increase in expression prior to D7 (Table S1). These results show that stratified epidermis in metamorphs requires an extended activation period (relative to paedomorphs) before migration begins and this delay contributes to a slower rate of re-epithelialization.

### Migrating Keratinocytes and Neoepidermis Express High MMP Levels

Epidermal expression and enzymatic activity of serine proteinases and matrix metalloproteinases has been shown to affect keratinocyte migration and ECM degradation during the early stages of mammalian wound healing [1]. In order to begin addressing the role these enzymes play during skin regeneration in axolotls we analyzed the expression of the serine proteinase *plasminogen tissue activator* (*PLAT*), matrix metalloproteinase (*MMP*) family members and the *tissue inhibitors of metalloproteinases* (*TIMP 1–4*) in migrating and neoepidermis following excisional wounding at D1, D3 and D7 in paedomorphs and metamorphs (Table S1). *PLAT* functions to convert plasminogen to plasmin and consistent with the absence of a fibrin clot was not expressed either in uninjured axolotl skin or following injury in the epidermis (Table S1).

**Figure 6:**
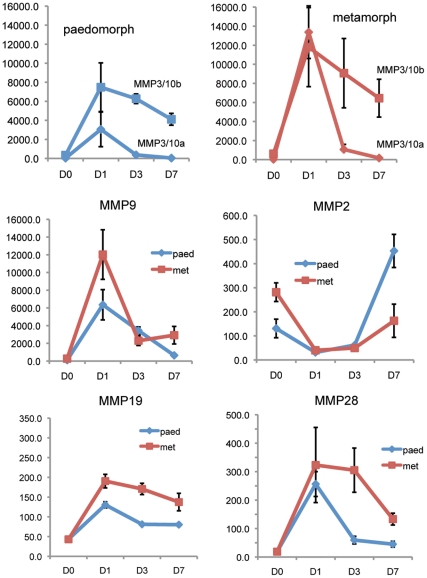
*Matrix metalloproteinase* (*MMP*) expression during re-epithelialization and new tissue formation. Expression values (y-axis) reflect absolute expression values from Affymetrix axolotl genechips (see Table S1 for exact values). Error bars represent standard error. Blue lines represent paedomorphs and red lines represent metamorphs. Expression kinetics for selected *MMPs* generally followed two patterns; (1) a strong upregulation at D1 followed by an equally strong decrease at D3 and a continued decrease or leveling off at D7 [*MMP3/10a, MMP9*] and (2) a strong upregulation at D1 which remained high at D3 and then downregulated at D7 [*MMP3/10b, MMP19*]. For *MMP28* the kinetics followed pattern 1 for paedomorphs and pattern 2 for metamorphs suggesting that *MMP28* expression was highly connected to re-epithelialization. The expression profile for *MMP2* was unique in that is was downregulated following injury and was upregulated after re-epithelialization was complete.

During skin regeneration in axolotls we found a strong *MMP* response to injury in both migrating keratinocytes and neoepidermis in paedomorphs and metamorphs (Table S1). For all *MMPs* demonstrating significant changes in gene expression following injury, the expression kinetics generally followed two patterns; (1) a strong upregulation at D1 followed by an equally strong decrease at D3 and a continued decrease or leveling off at D7 and (2) a strong upregulation at D1 which remained higher than baseline at D3 and then was downregulated at D7 (Figure 6).

The collagenases *MMP1* and *axCOL* (an amphibian specific collagenase orthologous to newt *nCOL*) followed the first pattern with *MMP1* exhibiting a particularly strong response, increasing 425-fold (paedomorphs) and 502-fold (metamorphs) at D1, while remaining 88-fold (paedomorphs) and 61-fold elevated (metamorphs) at D7 (Table S1). The stromelysin *MMP3/10a* exhibited a similar expression profile as the collegenases except D7 levels returned near baseline (Figure 6). *MMP3/10b* levels, while exhibiting strong upregulation at D1, exhibited a slow decreasing rate of expression (pattern 2) compared to *MMP3/10a* and remained elevated at D7 (Figure 6). While *MMP19* and *MMP28* followed expression pattern 1 for paedomorphs, expression of both genes remained high in metamorphs mirroring the lag time in the metamorphic re-epithelialization rate (Figure 6). The gelatinase *MMP9* exhibited pattern 1 expression kinetics and responded 2-fold greater in metamorphs. *MMP2* was the one exception to the generally observed patterns. *MMP2* was effectively turned off at D1, remained off at D3 and then was upregulated in both paedomorphs and metamorphs (Figure 6). Given the strong response to injury reflected in *MMP* levels, we also analyzed expression levels for the *tissue inhibitors of MMPs* (*TIMPs*) (Table S1). Only *TIMP1* changed significantly, with paedomorphs exhibiting pattern 1 expression and metamorphs exhibiting pattern 2. Interestingly, axolotls appear to have two copies of *TIMP1* based on sequence analysis and this second copy did not change in either morph following injury (data not shown). Previous experiments during newt limb regeneration have shown that *MMP* transcription correlates well with their enzymatic activity [34] and the high sequence similarity between newt and axolotl *MMP* sequences strongly supports MMP orthology. We conducted gelatin zymography during paedomorphic tail regeneration and found that transcription and activity of MMP9 were well correlated, thus supporting this association in axolotl (Figure S5 A-C). This confirms recent work during axolotl limb regeneration demonstrating the activity of axolotl MMP [35]. Taken together these results suggest MMPs may play a key role during keratinocyte migration and regeneration, and suggest that sustained activity may control the timing of ECM deposition.

### Metamorphic Axolotls Exhibit Increased Leukocyte Infiltration

**Figure 7:**
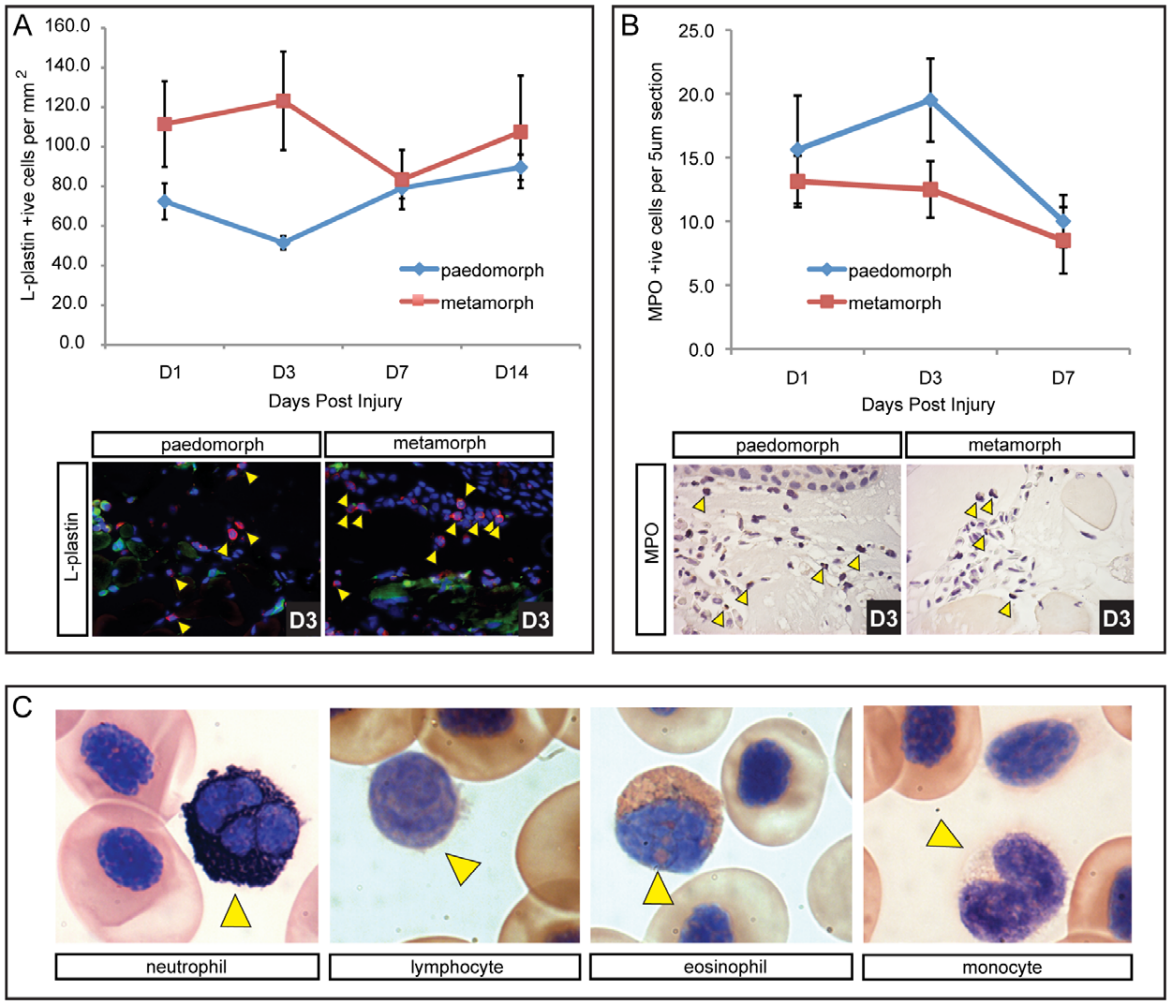
Higher initial leukocyte levels are present in terrestrial axolotls coincident with slower re-epithelialization, but neutrophil levels are not different between morphs. L-plastin was used as a pan-leukocytic marker to quantify total leukocyte levels following injury. A) Total leukocytes counted per mm^2^ in the wound bed at 1, 3, 7 and 14 dpi (n = 4 for each morph). L-plastin positive cells are red (yellow arrows), nuclei are stained blue (Hoescht) and green fluorescence was used to account for autofluorescencing erythrocytes that were excluded as leukocytes. An influx of leukocytes was observed 24 hrs dpi, with higher numbers present in terrestrial axolotls. Leukocyte levels dropped in metamorphs at D7 and converged with paedomorph levels. Levels for paedomorphs were not significantly changed after D1. B) All neutrophils present in the wound bed (yellow arrows) were counted on 5 µm sections above the muscle using myloperoxidase (n = 8 for each morph; see Figure S6 for positive control staining in liver). Neutrophil levels were generally low and were not significantly different between paedomorphs and metamorphs, although they did drop significantly at D7. C) Modified Wright-Giemsa stain used to indentify individual leukocytes in circulating axolotl blood. Sudan black was used to stain neutrophils. Yellow arrows indicate the specific leukocyte type.

During wound repair, adult mammals exhibit a stereotypical inflammatory response characterized by infiltration and subsequent removal of leukocytes [36]. The trade-off between regeneration and fibrosis has been postulated to result from a weak inflammatory response with greater inflammation contributing to increased fibrosis [37]. In order to characterize the inflammatory response during skin regeneration, we assayed the wound bed for L-plastin positive cells, a pan-leukocytic marker conserved in vertebrates. To control for individual variation we harvested wound tissue from the same animal (4 wounds per animal) at D0, D1, D3, D7 and D14 from paedomorphs (n = 4) and metamorphs (n = 4). Examining the number of L-plastin positive cells we found both morphs exhibited a robust infiltration of leukocytes into the wound bed (Figure 7A and Figure S6A). We used a 2-way ANOVA to examine the effects of morph (metamorph vs paedomorph) and day post injury on total leukocyte numbers (with individual as a random effect to allow for repeated sampling). This analysis yielded a small p-value for the effect of morph (F = 3.05, p = 0.095). An examination of each individual over time showed that one paedomorph exhibited much higher levels of L-plastin positive cells (suggesting chronic stress or sickness) compared with the other three individuals (Figure S6B, C; paed 4). Therefore we analyzed the data without this individual and found that metamorphs had a significantly higher number of leukocytes following injury (F = 5.32, p = 0.032) (Figure 7A). This difference was most pronounced between D1 and D3 (Figure 7A) when re-epithelialization was complete in paedomorphs but not in metamorphs.

In order to further quantify the inflammatory response we examined neutrophil infiltration in paedomorphs and metamorphs. Using a polyclonal antibody to myloperoxidase (MPO) we counted all neutrophils in 5µm sections of the wound bed between the epidermis and underlying muscle following injury (Figure 7B). Neutrophils were present in low numbers following injury (compared to mammals) and were not significantly different between paedomorphs (n = 8) and metamorphs (n = 8) (F = 2.50, p = 0.136). Examining MPO positive cells across all axolotls (n = 16) the effect of days post injury approached significance (F = 3.04, p = 0.058) and post-hoc comparisons revealed a significant decrease in neutrophil levels at D7 (t = 2.014, p = 0.0226).

In mammalian wounds, neutrophils represent the majority of leukocytes present during the early inflammatory response and comprise approximately 59% of circulating leukocytes [38]. Our data suggested that neutrophils comprised a relatively small fraction of infiltrating leukocytes in axolotls after injury, and we asked if this was due in part to low levels in circulation. Using a modified Wright’s stain to identify leukocytes and Sudan Black to accurately identify the neutrophil population, we calculated leukocyte profiles (percentage of total leukocytes) for both paedomorphs (n = 5) and metamorphs (n = 5) (Figure 7C and Table S2). We found that leukocytes comprise approximately 4% of circulating blood cells with neutrophils accounting for ∼21% of the leukocyte population in both forms (Table S2). Comparing paedomorphs and metamorphs we found significantly higher eosinophil levels in paedomorphs (t = 2.49, p = 0.043) and significantly higher lymphocyte levels in metamorphs (t = 3.96, p = 0.004). Taken together, these results show that axolotls display an influx of leukocytes following full thickness excisional wounding demonstrating an inflammatory response to wounding. However, relative to mammals, neutrophils comprise a smaller fraction of inflammatory cells at the wound site, and this correlates with their relatively lower representation in circulating blood.

### Percent Wound Closure Mimics Contraction Rates During Healing of Human Skin Wounds

**Figure 8:**
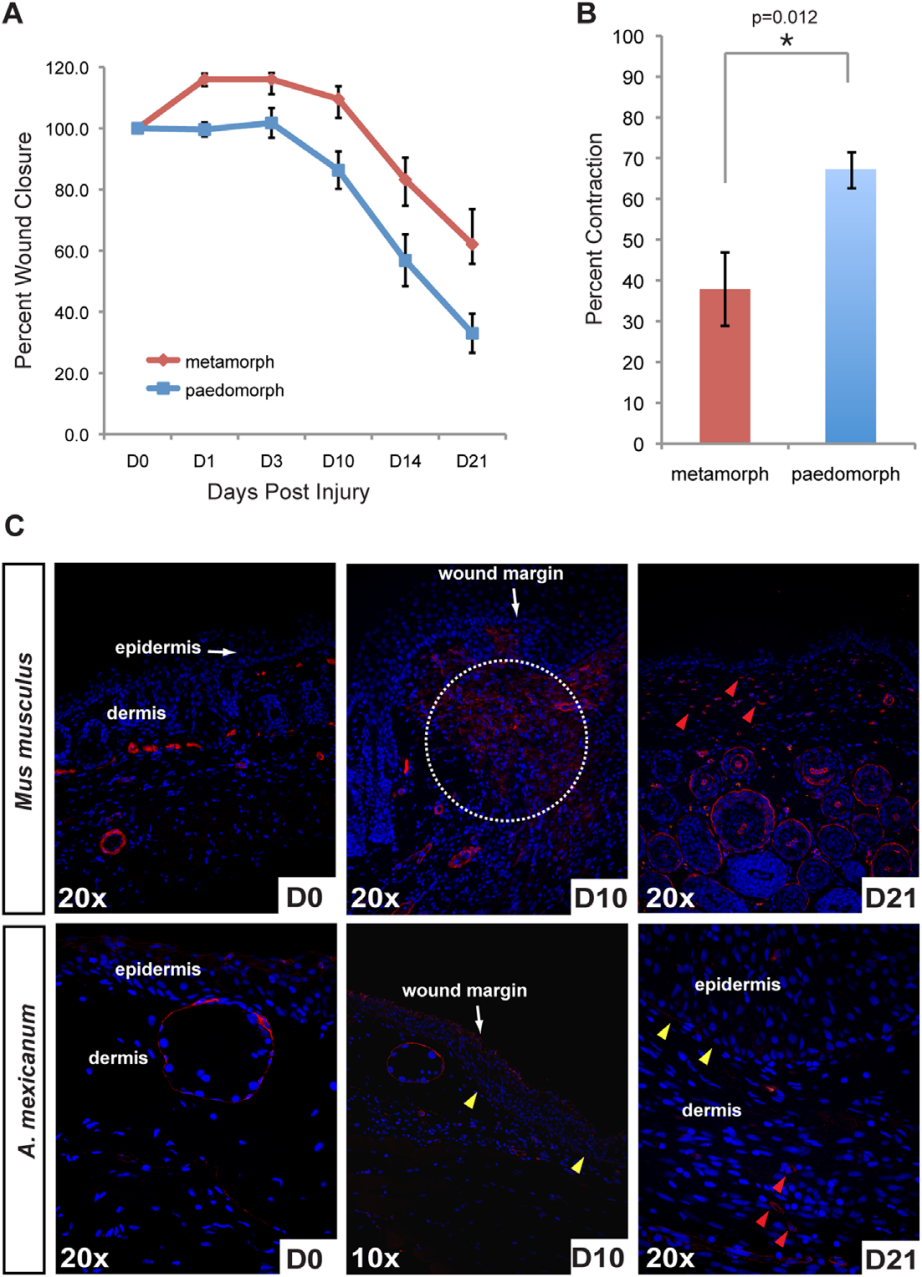
Contraction during scar-free healing in axolotls is similar to tight-skinned mammals. A) Percent wound closure in paedomorphs and metamorphs over 21 days (when contraction is complete). Metamorph wounds expanded by 10% following wounding and wounds contracted at about the same rate in both morphs. B) Paedomorph wounds contracted ∼27% more than metamorph wounds with contraction accounting for 37.9% of wound closure in metamorphs. C) Alpha-smooth muscle actin (alpha-SMA) localization in unwounded skin and during wound repair in *Mus musculus* and axolotls (*A. mexicanum*) to identify myofibroblasts. Alpha-SMA localized to blood vessels and a few cells in mouse skin and around glands and the stratum compactum in axolotl skin. Ten days post injury (dpi), when contraction rates are highest in mouse wounds, alpha-SMA positive cells are detected at high levels at the wound margins (white dotted circle). In contrast, we detected very few alpha-SMA positive cells ten days after wounding in axolotl tissue. We did, however, detect alpha-SMA in the regenerating basement membrane (yellow arrows). Twenty-one dpi we detected a high number of myofibroblasts in mouse tissue (red arrows). We detected a few myofibroblasts in axolotl tissue at D21 (red arrows) near the underlying muscle.

Wound contraction is an important component of the repair process and is an important metric when comparing wound repair across taxa [39]. In humans and pigs, contraction of circular wounds accounts for less than 50% of wound closure, while in rodents contraction accounts for ∼90% of repair [39]. Following excisional wounding, the wound area increased slightly in metamorphs while remaining constant in paedomorphs and after 24hrs the wounds in both groups contracted at approximately the same rate (Figure 8A). Quantifying the rate of wound contraction between paedomorphs and metamorphs, we found that percent contraction was significantly greater in paedomorphs and resulted in a greater degree of wound closure (paedomorphs = 67%±4.4% and metamorphs = 37.9%±9%) (Figure 8B). In fact, our analysis shows that metamorphic axolotls contract their wounds to approximately the same degree as human skin after wounding.

Prior to wound contraction, some dermal fibroblasts in the granulation tissue and at the wound margins begin to express smooth muscle actin and are responsible for generating contractile forces that pull the wound margins together [40], [41]. We also examined localization of alpha-smooth muscle actin (alpha-SMA) (a marker of myofibroblasts) in paedomorphs and compared its distribution to mice at similar time points after wounding (Fig. 8C). Approximately 10 days after injury, mice showed a high number of alpha-SMA positive cells at the wound margins when the wound area begins to rapidly contract (Figure 8C; white circled area). In contrast, we did not detect many alpha-SMA positive cells in axolotls at the wound margins or in the wound bed at D10, although the basement membrane beneath the wound epidermis appeared positive for alpha-SMA (Figure 8C). We observed numerous alpha-SMA positive cells at D21 in mouse granulation tissue compared with relatively few in axolotl provisional matrix. Taken together, these data show that wound contraction in axolotls mimic wound contraction in human skin, although there are relatively few myofibroblasts present during axolotl skin regeneration.

### Dermal regeneration is Characterized by High Levels of Tenascin-C

**Figure 9:**
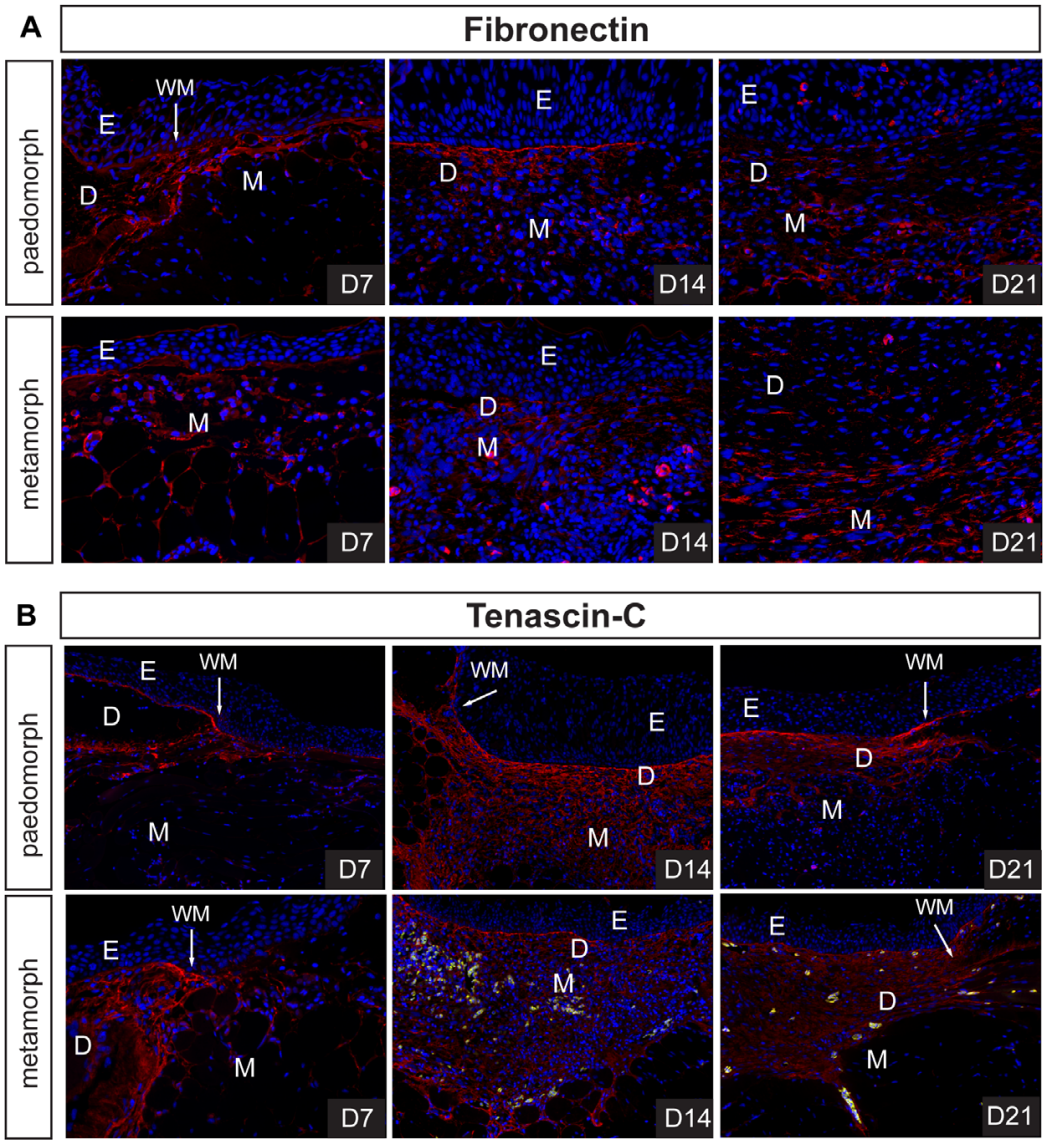
Regenerative ECM in axolotl wounds is characterized by high levels of tenascin-C. A-B) Fibronectin (FN) and tenascin-C (TN-C) levels were detected during scar-free healing in paedomorphs and metamorphs using an antibody to axolotl fibronectin and a polyclonal antibody to chick tenascin-C. We detected low levels of FN in the basement membrane at D7, and at the wound margins in both morphs. FN was present during ECM deposition at D14 in the center of the wound bed, but in relatively small amounts. By D21 little FN persisted in the regenerating dermis. B) TN-C was detected at the wound margins, in the basement membrane and surrounding some cells at D7. Fourteen days post injury we detected high levels of TN-C throughout the wound bed and in regenerating muscle. A sharp boundary formed between intact muscle and regenerating muscle. These high levels of TN-C persisted during dermis regeneration. Green fluorescence was used to detect autofluorescing erythrocytes. Epidermis (E), dermis (D), muscle (M), wound margin (WM).

**Figure 10:**
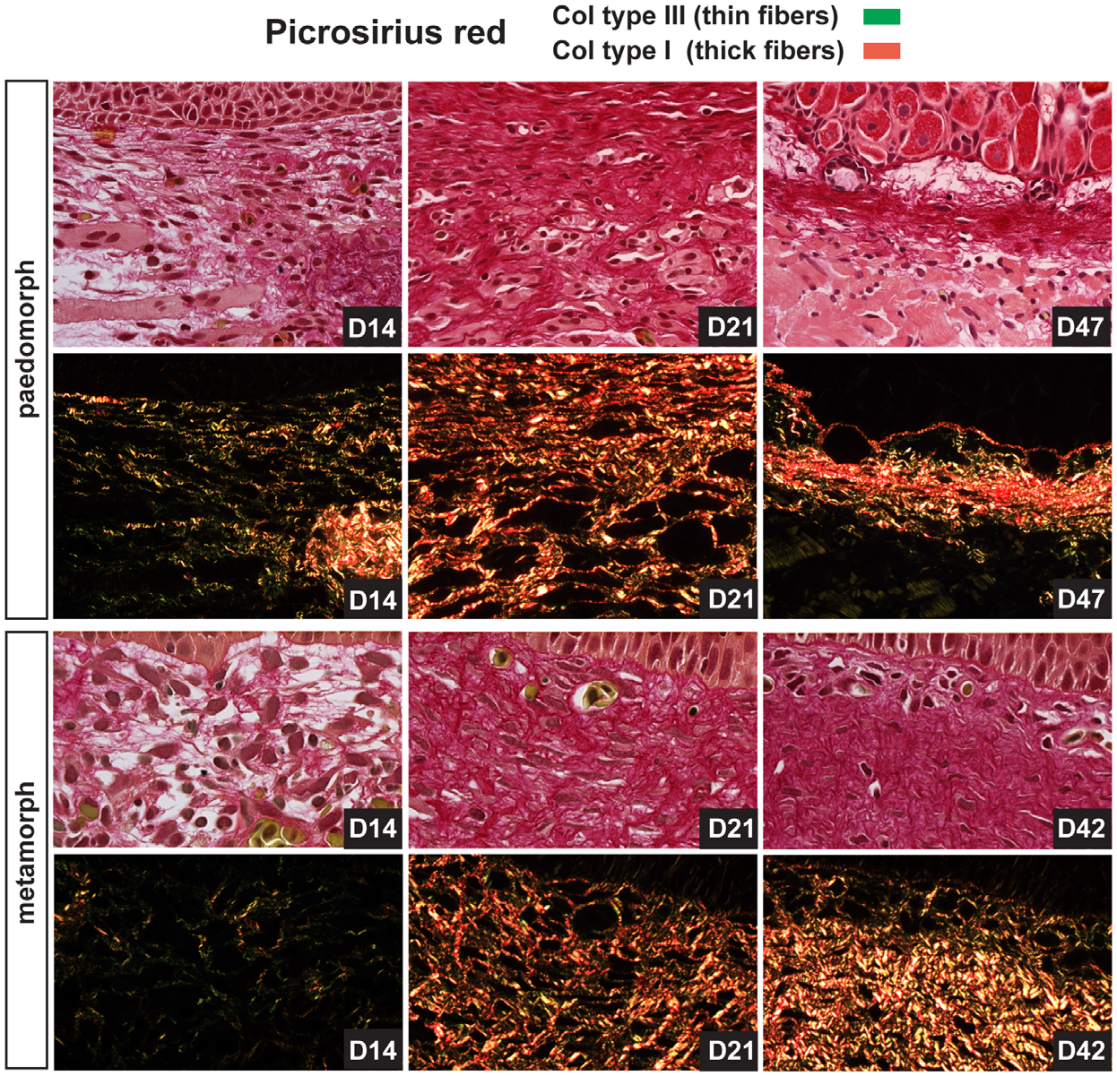
Collagen type III predominates early during new tissue formation and is slowly replaced by collagen type I during scar-free healing. Picrosirius red staining was used to detect collagen type III and collagen type I during scar-free healing in the wound bed. Using polarized light to detect bifringence, collagen type III (green fibers) was deposited first during new ECM deposition in both morphs. As the dermis regenerated, collagen type III was slowly replaced by collagen type I (red fibers) in both morphs.

The process of dermal repair in mammals (which ultimately results in a scar) proceeds via production of a fibrin clot, (referred to as the provisional extracellular matrix), degradation and replacement of provisional matrix by granulation tissue, and remodeling of granulation tissue into a fibrotic scar [1]. Following re-epithelialization in paedomorphs (D1) and metamorphs (D3), the wound bed was rich in blood cells and plasma but no scab formed (Figure 2). Provisional matrix in mammals is rich in fibronectin and thrombospondin [1] and along with plasma we detected low levels of fibronectin at the wound margins and beneath the wound epidermis at D7 in both morphs (Figure 9A). In contrast, we were unable to detect appreciable collagen deposition at D7 using histochemical techniques (Figure 1 and Figure 3). During mammalian wound healing, deposition of granulation tissue matrix proceeds in the stereotypical sequence of fibronectin, collagen type III and collagen type I [42]. At D14 fibronectin was present in low concentrations in granulation tissue in both morphs (Figure 9A). Using picrosirius red to detect both collagen type III and collagen type I, we predominantly observed collagen type III beneath the epidermis at D14 (Figure 10). As collagen synthesis continued collagen type III was replaced with collagen type I and as new dermis became progressively acellular, the density and diameter of collagen type I fibers increased (Figure 10). Fibronectin remained detectable in granulation tissue (D21), albeit at very low levels (Figure 9A).

While high levels of fibronectin and collagen are present in granulation tissue and also fibrotic scar tissue, the appearance of tenascin-C (TN-C) during mammalian wound healing is normally restricted to the wound margins and thought to stimulate cell migration. Concomitant with these observations we found intense TN-C protein localization at the wound margins and beneath the epidermis at D7 (Figure 9B). However, as dermal regeneration proceeded, TN-C persisted in high amounts throughout the wound bed and within regenerating muscle (Figure 9B). This association was particularly striking in the underlying muscle where TN-C formed a sharp boundary between regenerating and undamaged muscle (Figure 9B). TN-C levels remained high until the dermis had regenerated (Figure 9B). Taken together these findings suggest that collagen synthesis during axolotl scar-free healing proceeds similarly to that which is observed during mammalian wound repair and low levels of fibronectin and persistent high levels of TN-C characterize the anti-scarring matrix.

## Discussion

**Figure 11:**
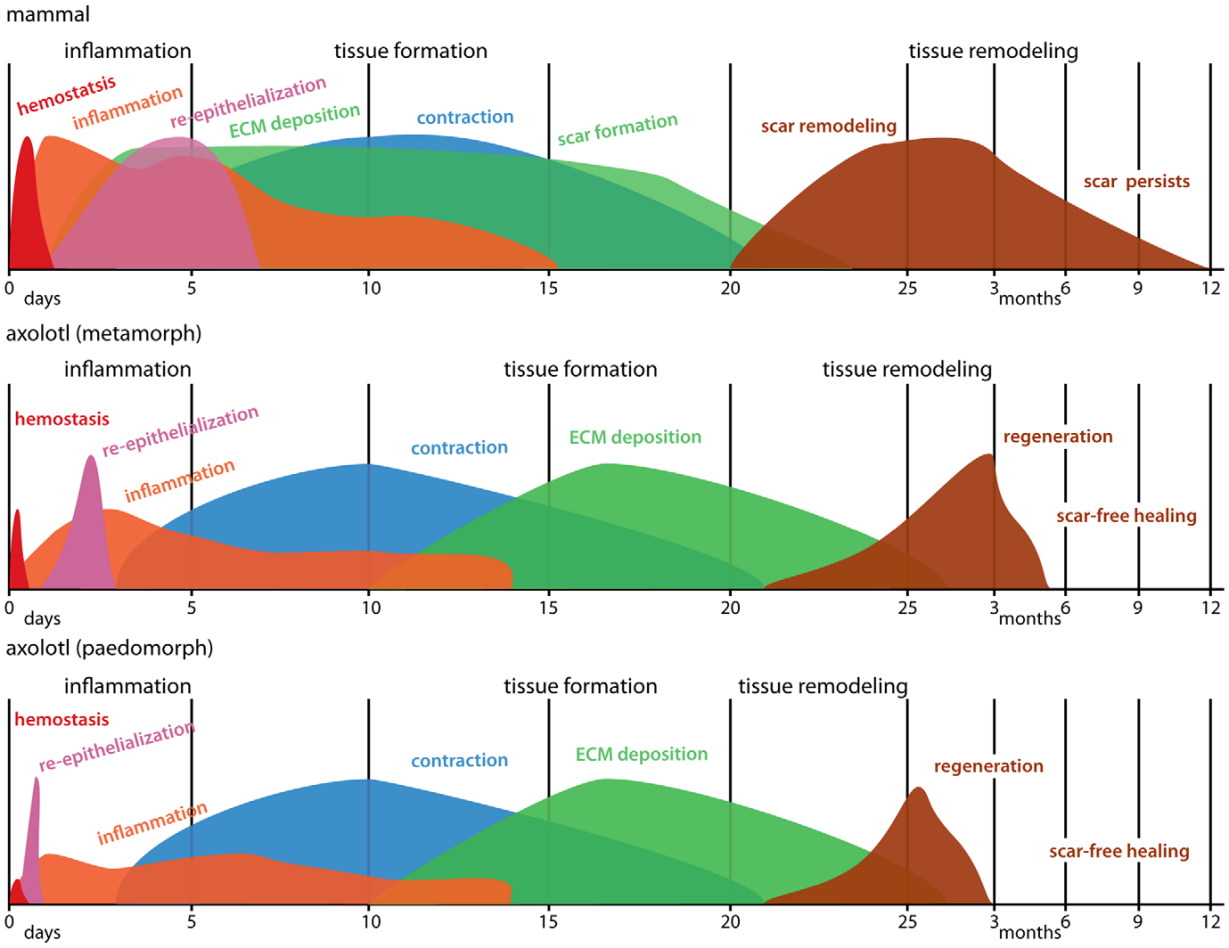
Summary of wound healing processes comparing axolotls and mammals. The x-axis represents time and the y-axis represents percent maximal response for each process. Information for mammals has been approximated from the literature and from our own experiments with 4mm FTE wounds in mice. Colors represent individual processes overlapping across the three phases of wound healing; inflammation, new tissue formation and tissue remodeling. Comparing paedomorphic and metamorphic axolotls, metamorphs exhibited an increased hemostatic response, slower re-epithelialization, increased early inflammatory response, increased and prolonged deposition of extracellular matrix (ECM) and an almost doubling in the time required for complete skin regeneration. Comparing scar-free healing in terrestrial axolotls to scar formation in mammals, terrestrial axolotls exhibited a reduced hemostatic response, lower neutrophil levels, faster re-epithelialization rate, delay in ECM production, differences in the relative composition of the new ECM, regeneration of glands and dermis regeneration instead of scarring. Schematic for mammals is adapted from Mikael Häggström.

Although many salamanders are capable of complete appendage regeneration (e.g. limb, tail), a recurring question is whether wounds made on the body heal with a scar or instead, heal scar-free [32]. In this study we demonstrate complete and perfect regeneration of adult axolotl skin following full thickness excisional (FTE) wounding to the dorsal flank. Additionally, we tested the hypothesis that loss of larval skin characters in metamorphic axolotls results in scarring following FTE wounding. Instead, we find that metamorphic axolotls are capable of scar-free healing. To our knowledge, this is the first demonstration of perfect scar-free healing of non-limb FTE wounds in an aquatic or terrestrial adult vertebrate. Comparing skin regeneration in paedomorphic (aquatic) and metamorphic (terrestrial) axolotls, metamorphic axolotls exhibited slower re-epithelialization, increased numbers of leukocytes during the early inflammatory response, increased deposition of extracellular matrix (ECM) and an almost doubling in the time required for complete skin regeneration. Compared to mammalian wound repair, terrestrial axolotls exhibited a reduced hemostatic response, lower neutrophils levels, similar duration of inflammation, faster time to complete re-epithelialization, a delay in new transitional matrix production and differences in the relative composition of the new ECM (Figure 11). These data suggest that further exploration of FTE wounding in axolotls is an excellent model to investigate the cellular and molecular regulation of scar-free healing.

### Hemostasis

Wound healing in mammals proceeds along a stereotypical timeline consisting of repair processes that overlap in time and space and these processes have generally been included within three general phases; inflammation, new tissue formation and tissue remodeling [1], [6], [43]. Wound healing begins immediately after injury through the extravasation of blood products and activation of the coagulation cascade. In mammals, subsequent hemostasis is achieved through formation of a thick clot formed from platelets and plasma derived fibrin. This clot acts as a temporary plug and as a reservoir of growth factors, while the fibrin matrix acts as a scaffold for infiltrating inflammatory cells. In aquatic axolotls, the hemostatic response appeared limited with a thin layer of coagulated plasma at the wound site that was mostly devoid of cells. Following re-epithelialization, the neoepidermis was in close proximity to the underlying muscle with little evidence of a persistent fibrin clot prior to new ECM production. The presence of a clot in terrestrial axolotls was more pronounced, but was still greatly reduced compared to mammals. Erythrocytes and plasma formed the majority of material above the fragmenting muscle and it was unclear to what extent fibrin contributed to the loosely aggregated clot matrix. Following re-epithelialization, some blood plasma, erythrocytes and leukocytes were observed above and within the fragmenting muscle fibers, but the extent of a clot remained minimal and the epidermis remained in close proximity to the underlying muscle. Taken together, these findings show that axolotls restore hemostasis quickly and without formation of an extensive fibrin clot.

During mammalian wound healing, platelets degranulate, initiate the coagulation cascade and provide a wealth of growth factors and chemokines (e.g. PDGF, TGFß, VEGF, EGF, and IGF) that activate mesenchymal cells and attract inflammatory cells to the wound site [7], [44]. Although some evidence suggests that platelets are not required during wound healing in mammals [45], all of these molecules have been shown to play a role during wound repair [46]. The degranulating action of amphibian platelets (thrombocytes) during injury is poorly understood, as are the growth factors produced during hemostasis. Future studies investigating the relative complement of chemoattractants and mitogens present in the clot matrix during scar-free healing in axolotls will shed light on how this molecular cocktail might direct the early events of wound healing towards regeneration in lieu of scarring.

### Inflammation

Including hemostasis, the sequential influx and action of neutrophils, macrophages and lymphocytes constitutes the inflammatory phase of wound repair. In mammalian wounds, neutrophils are the predominant cell type present immediately after injury and are necessary to destroy bacteria and combat infection. The recruitment of neutrophils occurs passively (through plasma aggregation) and via chemotaxis in response to the activation of complement, degranulating platelets, and from bacterial degradation. Neutrophils in turn have the capacity to attract additional inflammatory cells and amplify the inflammatory response. Monocytes arrive at the wound site 2–3 days after injury and transform into macrophages. Macrophages serve to remove neutrophils and cellular debris, which helps to resolve inflammation, and also are thought to assist later during new tissue formation to regulate the balance between fibrosis and scarring [36]. Following FTE wounding in aquatic and terrestrial axolotls we detected neutrophils 24 hrs after injury. Neutrophil levels, however, were reduced compared to equal size FTE wounds in mammals suggesting a reduction through passive aggregation or chemotaxis [47]. Compared to mammals, which maintain ∼60% of their circulating leukocytes as neutrophils, we found axolotls maintain approximately ∼21% of their circulating leukocytes as neutrophils and this suggests that low neutrophil numbers at the wound site result partly from low numbers in circulation. In support of this finding, a recent study in larval axolotls found very few neutrophils in partial thickness tail wounds (which incur very little bleeding), while stab wounds made into the tail muscle, led to greater numbers at the wound site [26]. Neutrophil depletion studies in mammals have shown that as long as conditions remain sterile, neutrophils are not required during wound healing, and their loss may actually increase the rate of re-epithelialization [48], [49]. Our findings show that their presence, albeit in low numbers, is compatible with scar-free healing and is coincident with a faster rate of re-epithelialization compared to mammalian wounds.

Although we did not examine additional individual leukocytic lineages, comparing L-plastin positive cells (a pan-leukocytic marker) in aquatic and terrestrial axolotls, we found higher total leukocyte numbers persisting in terrestrial animals while the wound bed remained exposed. Following re-epithelialization, total leukocyte numbers converged for both morphs suggesting that the early influx of inflammatory cells may not significantly influence the subsequent arrival of inflammatory cells. Together, our finding that total leukocyte levels remain high at least 14 days post injury supports the role of an active inflammatory phase during scar-free healing in axolotls.

The notion that reduced inflammation promotes regeneration in lieu of scarring has been popular, but evidence from mammals and amphibians remains inconclusive [50], [51]. Aspects of limb regeneration in adult anurans, which lose the ability for limb regeneration following metamorphosis, can be stimulated through tissue aggravation [52] and chemical irritation [53], suggesting that prolonging tissue inflammation can prime a dormant regenerative response. On the other hand, scar-free healing in fetal mammals is correlated with reduced inflammation prior to their development of a mature immune system (compared to scarring in adults) [10], [54]. Data from adult mammals suggests platelets [45], neutrophils [48] and macrophages [55] are dispensable for wound repair, but their removal does not lead to scar-free healing suggesting that reducing inflammation alone will not induce a regenerative response. The existing data from chronic and hypertrophic wounds support the hypothesis that too much inflammation promotes excessive fibrosis, and in the context of our results, it is likely that inflammation must not exceed a threshold level for scar-free healing to occur [7], [56]. A recent study designed to increase inflammation in axolotls by injecting bleomycin following partial thickness tail wounds, found evidence of increased fibrosis following thirty days of administering the drug during the healing process [26]. While it is tempting to speculate that this exposure might lead to scarring, given the continuous exposure to bleomycin prior to tissue harvest it is difficult to interpret their results as they relate to heightened inflammation early during the healing process. Future experiments over-stimulating inflammation in our terrestrial FTE model of scar-free healing through chemical and molecular methods during the naturally occurring window of inflammation will allow us to examine the cellular and molecular mechanisms that connect inflammation and fibrosis. Our findings reinforce the idea that inflammation is not altogether anti-regenerative and support a role for an active inflammatory response during scar-free healing.

### Re-epithelialization

As the inflammatory phase begins to wind down, keratinocytes at the wound-edge migrate to re-epithelialize the wound bed and restore the physical barrier separating the external environment. Concomitantly, granulation tissue (transitional matrix) is produced to replace the fibrin clot and together these processes will contribute to new tissue formation at the wound site. We found that adult paedomorphic axolotls completely re-epithelialized 4mm wounds 18–24 hrs post injury, while terrestrial axolotls exhibited slower re-epithelialization. In terrestrial axolotl skin, wound edge keratinocytes migrated after a delay period of ∼24 hrs and complete re-epithelialization occurred up to 3 days after injury. This finding suggests that the transition from a pseudo-stratified to stratified epidermis following metamorphosis prevents rapid re-epithelialization in response to injury. Previous research in newt limbs suggested that epidermal keratinocytes are constitutively active and thus are primed to migrate following injury [24]. Our finding in paedomorphs supports this conclusion, while the observed delay in terrestrial axolotls demonstrates the need for keratinocyte activation as occurs in mammalian wound healing [57]. Interestingly, the onset of keratinocyte migration in mammals occurs after a delay period of 18–24 hrs [57], [58], which is identical to what we observe in terrestrial axolotls. FTE wounds made in fetal rats that heal scar-free (E16), or result in scarring (E19), both re-epithelialize in 72hrs and together with our data in terrestrial axolotls, suggest that the speed of re-epithelialization cannot alone explain the ability to perfectly regenerate skin [13]. Our results suggest that rapid re-epithelialization reduces the time required to regenerate skin, and may contribute to reduced inflammation. Once activated, terrestrial axolotl keratinocytes appeared to migrate rapidly across the wound bed and it would be interesting to compare the rate of re-epithelial migration to similar sized mammalian wounds where moisture prevents scab formation. Comparing epidermal cells between these two morphs during wound healing will provide a powerful model to identify the molecular control of keratinocyte activation and will be useful in identifying new molecules that might stimulate keratinocyte migration in non-healing chronic wounds.

### ECM Production and New Tissue Formation

While a dampened hemostatic response, reduced inflammation and quicker rate of re-epithelialization likely contributes to scar-free healing in axolotls (compared to scarring in adult mammals), the slow deposition of new extracellular matrix (ECM) and its unique molecular composition suggests newly synthesized axolotl ECM may antagonize fibrosis and promote regeneration (Figure 11). Shortly after re-epithelialization begins during mammalian wound repair, the provisional matrix, which is rich in cross-linked plasma fibronectin, is replaced by granulation tissue rich in macrophages, fibroblasts, newly synthesized ECM molecules and neovasculature [59]. Within this granulation tissue, fibroblasts secrete fibronectin, collagen type III and collagen type I, and this matrix will form the transitional ECM that will ultimately be remodeled into dermal scar tissue [1], [60]. Although fibronectin and collagen are the most abundant ECM molecules in mammalian granulation tissue, tenascin-C is also deposited, first at the wound margins, then at the epidermal-dermal junction of migrating keratinocytes, and finally, within the granulation tissue itself [61]. During scar-free healing in terrestrial axolotls the wound bed at the epidermal-dermal junction contained little new ECM for 10 days after re-epithelialization. This delay period was striking in its duration and our finding that the neoepidermis was a potent source of several matrix metalloproteinases (MMPs) suggests an active inhibition of new ECM production. While we found strong and rapid upregulation of several *MMPs* (*MMP1, axCol, MMP3/10a,b, MMP19, MMP28, MMP9*) prior to, and during re-epithelialization, most of these returned near baseline levels following re-epithelialization supporting their role during keratinocyte migration [25]. However, *MMP3/10b* and *MMP9* persisted at relatively high levels in the neoepidermis, and *MMP2* levels began increasing after re-epithelialization. All three of the homologous mammalian proteinases can degrade fibronectin, and MMP3 and MMP2 are capable of degrading collagen type III and type I [62], [63]. Together these data suggest that during scar-free healing in axolotls, persistently high MMP levels may act after re-epithelialization to prevent new ECM production as resident tissue undergoes histolysis and the wound bed is prepared for deposition of the regenerative matrix. Supporting this notion, broad inhibition of MMPs during newt limb regeneration can lead to acellular scar tissue formation and studies during scar-free healing in fetal wounds have observed higher MMP levels compared to adult wounds [13], [34]. Ongoing studies in our laboratory are targeting these individual MMPs and their specific activities towards understanding how they regulate both re-epithelialization and the fibrotic response during scar-free healing.

The role of other proteinases such as the ADAMs and ADAMTSs during mammalian wound healing is poorly understood, while their role during fetal wound healing is completely unknown. Their ability to cleave membrane-bound domains of a host of different proteins (e.g. cytokines, growth factors, cytokine and growth factor receptors) thus releasing them into the extracellular space, has driven speculation that these factors may play an important role during wound healing, although direct studies are lacking [64]. Only ADAM9 and ADAMTS1 have been found to have a direct role during wound healing, with faster re-epithelialization occurring in mouse knockout studies of both individual genes [65], [66]. During scar-free healing in axolotls we found a small but significant increase in *ADAM9* following injury but did not detect changes above baseline in any of the other *ADAM* or *ADAMTS* genes. Together, our findings suggest that ADAMs may not play a vital role during scar-free healing, although they are likely to work synergistically during keratinocyte migration.

Comparing our ECM data with that acquired from studies on scar-free healing in fetal mammals, both similarities and differences can be noted. Although there has been considerable controversy surrounding the production of collagens in fetal wounds, owing to the variety of species, wound models and fetal age, it is generally accepted that collagens are produced during fetal wound healing and are produced more rapidly compared to adult wounds [67], [68]. While collagen fibrils are deposited in dense bundles parallel to the wound bed, fetal collagen fibrils appear to be deposited in a reticular fashion, almost identical to the surrounding dermis [68]. This contrasts with the situation during scar-free healing in axolotls where collagen deposition was delayed (as in adult mammals) and also occurred in dense bundles throughout the wound bed. Thus our data suggests that the pattern of collagen deposition observed during adult scarring is not technically incongruent with regeneration and may suggest the lack of later remodeling events or differences in the structural composition of the collagen deposited.

The appearance of new ECM during axolotl wound healing was coincident with fibroblast infiltration beginning at D14, and small amounts of fibronectin production. Similarly, fibronectin is recognized as a prominent component of the provisional matrix during adult and fetal wound healing, appearing early associated with the fibrin clot and then slowly disappearing concomitant with the deposition of collagen [60], [68]. It is expressed earlier in fetal wounds compared to adult wounds and its disappearance is correlated with the onset of collagen deposition. It is also present at high levels in unwounded fetal skin compared to adults [69]. In contrast to mammalian wounds, axolotl transitional matrix was highly enriched with tenascin-C. Fibronectin appeared transiently with collagen type III in the wound bed while tenascin-C persisted through D42 as collagen type I was produced and the dermis regenerated. The role of tenascin-C during fetal wound healing has been poorly studied, although in the same study that examined fibronectin distribution during incisional healing in the lip [68] tenascin-C levels were found to be slightly higher in the fetal versus the adult. Some data suggests that tenascin-C can antagonize certain functions of fibronectin including T-cell activation [70] and in the context of fetal scar-free healing and axolotl scar-free healing suggests that the relative concentration of each in the extracellular matrix might act to regulate an adaptive immune response. Tenascin-C is thought to behave as an anti-adhesive molecule promoting proliferation and migration over differentiation and as such, is a transient molecule during mammalian wound healing [1]. Tenascin-C can disrupt cell-matrix interactions and has been speculated to maintain monocytes and blastemal cells in a de-differentiated state during salamander limb regeneration [23], [71], [72]. These data suggest that the composition of ECM produced during a regenerative response may have an anti-fibrotic effect that helps to recapitulate embryonic development and promote a regenerative response.

### Remodeling and Regeneration

The evolutionary process that has led to wound repair while efficient, is not functionally perfect. Epidermal appendages do not regenerate and the uninjured dermal architecture is replaced by dense parallel bundles of collagen that reduce the mechanical properties of normal skin. While the wound bed ECM during mammalian wound repair will eventually result in a fibrotic scar, we observed complete regeneration of not only dermal layers but of epithelial derived glands. The regeneration of hair follicles has been observed in rabbit wounds and ear punches and in very large wounds made on young mice [73], [74], [75]. Other than these reports, the regeneration of epithelial-derived structures in adult vertebrates has not been observed. Although hair follicle and gland development is well understood in mammals, it is poorly understood at the molecular level in amphibians. Ongoing experiments in our laboratory examining the molecular control of gland development are underway and it will be interesting to determine how regeneration of these structures is controlled following scar-free healing [76]. Specifically, it will be of interest to determine the location of inductive signals that lead to the specification of new glands and whether these signals are localized or ubiquitous during the regenerative process.

### Axolotl Model of Scar-free Healing

The data in this paper establishes a novel, adult model of perfect skin regeneration in which to test hypotheses about the molecular regulation of fibrosis and more generally, the underlying genetic control of scar-free healing. As such, we have developed an FTE wound model in both aquatic and terrestrial axolotls and characterized their ability to perfectly regenerate all damaged tissues. To date no *adult* animal model has been described that is capable of perfectly healing FTE wounds and underlying muscle (outside of the limb and tail). While numerous studies have described healing without scarring in fetal mammals and marsupials [10], the fetal model provides a number of drawbacks that the axolotl model overcomes. First, the fetus is still developing in the womb and this creates both biological and practical complications. The developing fetus, at the time when it heals scar-free, has an immature endocrine system, is immuno-incompetent, is contained in a moist sterile environment, and its cells are in a state of chronic hypoxia [16]. In contrast to fetal models, terrestrial axolotls are fully developed, sexually mature, have a fully developed immune system (albeit not as sophisticated as adult mammals), and their FTE wounds are desiccating and open to infection. From a practical standpoint, “in utero” surgery is not required, up to six 4 mm FTE wounds can be made on the dorsal skin and pharmacologic drugs and chemicals can be easily delivered to antagonize or agonize signaling pathways. In combination with their ability to perfectly regenerate all tissues damaged in these wounds (e.g. epidermis, dermis, glands, nerves, muscle) this model shifts experimentation into a system where the ultimate clinical outcome actually occurs.

This new animal model will provide investigators a new paradigm in which to address the multiple processes of wound repair (e.g. hemostasis, inflammation, keratinocyte activation and migration, ECM formation, tissue remodeling) in a truly scar-free healing adult, while also providing a tractable genetic system to investigate the cellular and molecular mechanisms that regulate these processes. Given the considerable body of literature on the molecular pathways and cellular processes involved during normal wound repair, understanding how these conserved molecular players operate in a pro-regenerative environment will lead to development of new therapies that target downstream effectors for testing in established mammalian models.

## Materials and Methods

### Animals and Wound Model

#### Ethics Statement

All procedures were conducted in accordance with, and approved by, the University of Florida Institutional Animal Care and Use Committee (IACUC Study #201101534 and #200903505).

#### Animals

*Ambystoma mexicanum* (Mexican axolotl) were acquired from the Ambystoma Genetic Stock Center (AGSC, Lexington KY) and bred in captivity. Animals were housed individually in Aquatic Habitats® (Aquatic Habitats Inc., Apopka, FL) flow-through systems at 21–23°C in Holtfreter’s Solution and maintained on California blackworms (*Lumbriculus sp*., J.F. Enterprises, Oakdale, CA). Animals were deemed adults after reaching sexual maturity (>9 months) and measured between 12–15 cm total length. Metamorphic (terrestrial) axolotls were acquired either directly from AGSC or transformed at the University of Florida through treatment with thyroxine [77]. In both cases metamorphosis occurred after animals had reached sexual maturity. Terrestrial axolotls were housed individually in 10-liter containers containing moist paper towels and fed nightcrawlers. Mice used in this study were Swiss Webster (Charles River) approximately 6-months old.

#### Full thickness excisional (FTE) wounding

Adult axolotls were anesthetized by full submersion in Benzocaine (Sigma) 0.01% (aquatic) or 0.02% (terrestrial). Sterile 4mm biopsy punches were used to create FTE wounds through the skin into the dorsal back muscle. In this way, 6 wounds were created on the dorsal surface posterior to the forelimbs and anterior to the hindlimbs. In order to harvest wound tissue at specific time-points, animals were anesthetized as above and the entire wound was harvested using a 6mm biopsy punch and iridectomy scissors. FTE wounds in mice (through the panniculus carnosus) were made with 4mm biopsy punches through the dorsal skin posterior to the forelimbs and anterior to the hindlimbs.

### Histology and Immunohistochemistry

For histological and immunohistochemical analysis, harvested tissues were fixed in 10% NBF at 4°C for 16–24hr, washed in PBS and dehydrated to 70% EtOH. Samples were stored at 4°C and processed for paraffin embedding. Samples were cut at 5µm. For frozen sections, samples were fixed for 1hr in 10%NBF at 4°C, washed in PBS, equilibrated in sucrose and OCT freezing medium and frozen in OCT. Frozen sections where cut at 12µm. For histological analysis, paraffin sections were stained with one of the following: Masson’s Trichrome (Richard Allen Scientific) or Picrosirius Red (American Mastertech). For blood smears, blood was collected from limb amputations and air-dried on slides. Slides were stained with Sudan Black [78] to detect neutrophils and counterstained with a modified Wright-Giemsa Stain (Kodak).

#### Immunohistochemistry

Slides were rehydrated, antigen retrieval performed if necessary (see below for each antibody), washed in TBS, blocked for streptavidin and biotin (Vector Labs), and incubated with 1° antibody (see below) overnight at 4°C. The following day, slides were washed in TBS, incubated with biotinylated 2° antibody (Vector Labs) and subsequently incubated with streptavidin conjugated horseradish peroxidase antibody (Vector Labs) and visualized using DAB (3,3–diaminobenzidine) (Vector Labs) or incubated with a streptavidin conjugated Alexa-Flour 594 (Invitrogen). Images were captured on a Nikon Eclipse 6600 upright compound microscope using a Cool-Snap Pro true color camera (light microscopy) or an Olympus inverted microscope (IX81) with fluorescence using a Leica DFC310 FX (fluorescence microscopy).

#### Antibodies

For paraffin sections; Widespectrum Cytokeratin (DAKO; Z0622) 1∶500, *Ambystoma* Fibronectin (gift of Thierry Darribere) 1∶1000, Tenascin-C (Millipore, AB19013) 1∶500, Collagen type IV (Rockland, 600-401-106-0.1) 1∶450, alpha-smooth muscle actin (Abcam, ab5694) 1∶100, Laminin (DAKO, Z0097) 1∶500. For frozen sections; Myloperoxidase (Thermo, RB-373-A) 1∶100, L-plastin (gift of Paul Martin) 1∶2000. Antigen retrievals used were; microwave and Citrate Buffer (pH. 6.0) (Cytokeratin, Myloperoxidase, L-plastin, Tenascin-C, alpha-smooth muscle actin) or Proteinase-K treatment (DAKO) for 2 mins (Collagen type IV, Fibronectin, Laminin). Negative controls used isotyped IgG at the same concentration as the 1° antibody. A GFP filter was used to detect autofluorescence of erythrocytes.

### Microarray Analysis

Epidermal tissue was harvested using a 4mm biopsy punch. Two wounds were made along the flank and posterior to the forelimbs. Harvested epidermis was pooled for each animal. Four biological replicates were collected from uninjured epidermis (D0) and at 1, 3, and 7 days post injury. RNA was isolated using Trizol Reagent (Invitrogen) followed by Qiagen RNeasy Clean-up columns (Qiagen). RNA quality was assessed on an Agilent 2100 Bioanalyzer (Agilent). RNA processing for Affymetrix microarray analysis was performed at the University of Kentucky Microarray Core Facility and hybridized to 32, AMBY_002a520748F 2^nd^ Generation Axolotl Affymetrix microarrays (Affymetrix) [79]. Microarray data was tested for quality control and analyzed in the software system R using the Bioconductor package and oneChannelGUI [80], [81], [82]. Microarray data was preprocessed, and normalized using RMA and a linear model testing the effect of time for each morph type was fit for each gene across all 32 GeneChips using limma [83], [84]. Thus, two gene lists were produced containing significantly changed genes over time in paedomorphic or metamorphic animals. In these analyses, contrasts were made between each time point and a t-statistic was produced for each contrast. The moderated F-statistic calculated by the eBayes limma function [85] was used as an overall test for significance across time, similar to ANOVA. A gene was considered statistically significant if it had an FDR adjusted p-value <0.05 and changed at least 2 fold between any two time points. The microarray data is MIAME compliant and the CEL files are available on Sal-Site (www.ambystoma.org). In addition, the raw data is available through the public NCBI GEO database (accession number: GSE35255).

### Statistics

For quantification of inflammation we used a 2-way ANOVA to examine the effects of morph (metamorph versus paedomorph) and day post injury on total leukocyte numbers (with individual as a random effect to allow for repeated sampling). For all other statistical analyses we used a Student’s T-test.

## Supporting Information

Figure S1 **Axolotl (paedomorph) skin glands.** A) Granular gland. B) Mucous gland.
(TIF)

Figure S2 **Detailed aspects of dermis and gland regeneration.** A) High magnification image of a gland regenerating and descending from the epidermis 44 dpi. B) Detail of wound margin (WM) showing the edge of the injured stratum compactum which is normally densely compacted and the loose collagen fibers beginning to coalesce in the wound bed. C) Detail of wound bed 180 dpi showing mature mucous gland and regenerated stratum compactum. Hypodermis (H) and dermis (D). Scale bars  =  100 µm.
(TIF)

Figure S3 **Detailed aspects of metamorphic axolotl skin regeneration over 147 days.** A) Granular and mucous glands (yellow arrows) present in the stratum spongiosum. Collagen fibers are present between the glands. B) Some cellular aggregations in the epidermis appear to be early stages of regenerating glands (yellow arrows). C) Regenerated glands within densely compacted collagenous ECM beneath the epidermis. D) Stratum compactum is beginning to coalesce as the rest of the dermis has regenerated. Some fibrotic tissue remains within the regenerated muscle. E) Complete scar-free skin regeneration at D147 dpi. All tissue layers are present. Granular glands remain immature compared to uninjured skin. Scale bars = 100 µm.
(TIF)

Figure S4 **Visualizing the rate of re-epithelialization in paedomorphic axolotls using GFP transplanted skin.** 1.5 cm×1.5 cm squares of dermis and epidermis from ubiquitously expressing GFP axolotls were transplanted to same size explanted areas on the tail of adult white axolotls. After transplants had healed 4mm FTE biopsy punches were made through the transplanted tissue and GFP-labeled epidermis was observed and photographed migrating to cover the wound bed over 18hrs. Migration was observed beginning 3hrs post injury and was complete between 18 and 24hrs.
(TIF)

Figure S5 ***MMP*** **transcription and activity are correlated during tail regeneration in paedomorphs.** A) Axolotl *MMP9* expression (semi-quantitative PCR) from tail tissue following amputation and through D14 post-amputation. Expression is downregulated 7 days post injury. B) Gelatin zymography was used to assess MMP activity over 21 days post injury. Red arrow points to tentatively assigned MMP9 position based on size (∼85 kDa) from previously published work in newt and axolotl (Vinarasky et al. 2005, Santosh et al. 2011). C) Gelatinase activity lags behind transcriptional upregulation and peaks at D7. For quantification of activity, gel images were inverted, mean optical density of individual black bands were calculated on an Gel Logic gel imaging system (Kodak) and light was blocked to calculate a baseline (black) reference for each measurement. Standard errors are reported. Regenerating tail tissue and tissue 0.5mm rostral to the amputation plane was collected at 0hrs, 3hrs, 6hrs, 12hrs, 24hrs, 7 d, 14 d, and 21 d after injury (n = 3 per time point), snap frozen on dry ice, and stored at –80°C. Tissues were homogenized for 10 minutes on ice in 1:4 (w:v) homogenization solution [50 mol/L Tris-Cl (pH 7.6), 150 mol/L NaCl, 1% (v/v) Triton-X100, 10 mol/L EDTA, 1 mol/L PMSF], sonicated, set on ice for 10 minutes, and spun at 14 000×*g* for 10 minutes at 4°C. Supernatants were decanted off, quantified using a BCA protein assay kit (Pierce), and stored at –80°C. 25 µg total protein was diluted in zymogram sample buffer (Bio-Rad) and electrophoresed on Ready Gel zymogram polyacrylimide gels containing gelatin (Bio-Rad). MMP proteins were re-natured by washing gels in zymogram renaturation buffer (Bio-Rad) for 30 minutes and incubated for 16 hrs at 37°C in development buffer (Bio-Rad). Gels were stained with 0.5% (w/v) Coomassie Blue R-250 (Bio-Rad) for 30 minutes and de-stained with an acetic acid, methanol, and dH20 solution (1∶5∶4) until clear bands were visible.
(TIF)

Figure S6 **Individual variation in leukocyte numbers during scar-free healing.** A) Total leukocyte numbers based on L-plastin staining for paedomorphs and metamorphs (n = 4 for each morph). Because multiple wounds were made on the same animals the inflammatory response could be tracked per individual. B) One paedomorph exhibited an unusually high inflammatory response (paed 4) and we removed it from our analysis. C) Variation across metamorphs revealed no outliers. D) Control staining for myloperoxidase in axolotl liver section.
(TIF)

Table S1 **Axolotl gene identifications based on first homologous human gene hit in BLAST.** Expression values reflect absolute expression on genechip (max expression value = 30 k; expression values <100 = no expression). N = 4 animals per time point. D0P = day 0 paedomorph, etc. D0M = day 0 metamorph.
(XLS)

Table S2 **Leukocyte profiles for** ***Ambystoma mexicanum*** **(paedomorph and metamorph).** Numbers for each particular leukocyte type represented as a percentage of total leukocytes (thrombocytes were not counted).
(DOC)
