# Supplementary figures and images for: Skin Regeneration in Adult Axolotls: A Blueprint for Scar-Free Healing in Vertebrates
Source: PLoS One. 2012 Apr 2;7(4):e32875. doi: 10.1371/journal.pone.0032875 (PMC3317654; doi:10.1371/journal.pone.0032875)

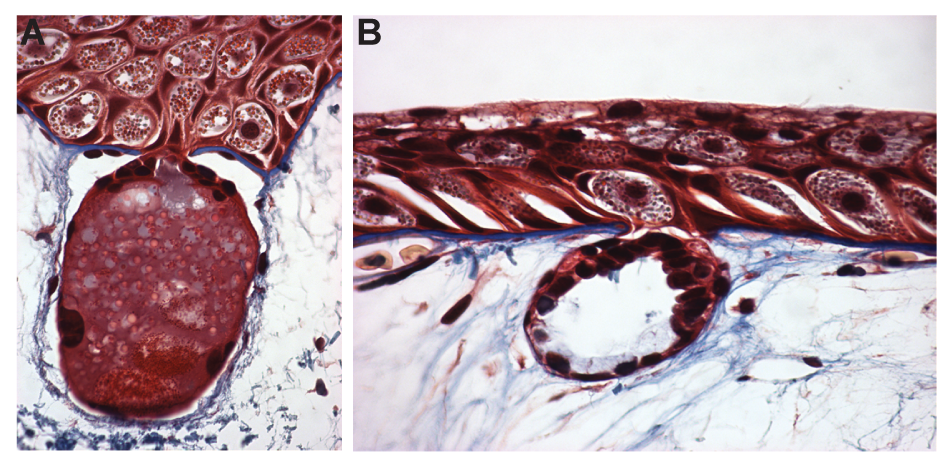

Supplement: Figure S1 — Axolotl (paedomorph) skin glands. A) Granular gland. B) Mucous gland. (TIF) [file pone.0032875.s001.tif]

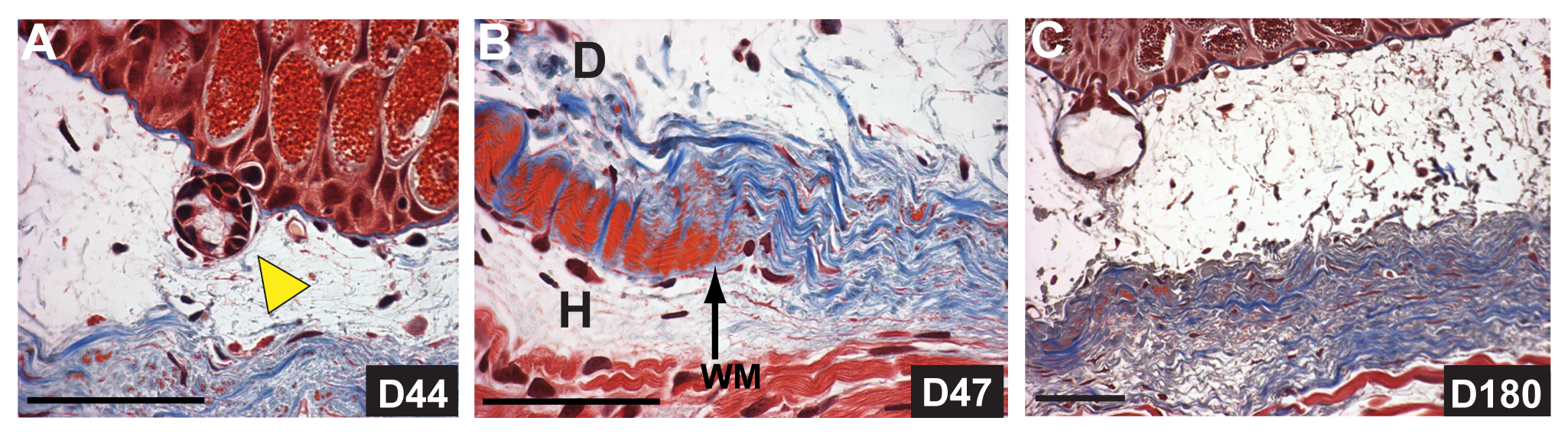

Supplement: Figure S2 — Detailed aspects of dermis and gland regeneration. A) High magnification image of a gland regenerating and descending from the epidermis 44 dpi. B) Detail of wound margin (WM) showing the edge of the injured stratum compactum which is normally densely compacted and the loose collagen fibers beginning to coalesce in the wound bed. C) Detail of wound bed 180 dpi showing mature mucous gland and regenerated stratum compactum. Hypodermis (H) and dermis (D). Scale bars = 100 µm. (TIF) [file pone.0032875.s002.tif]

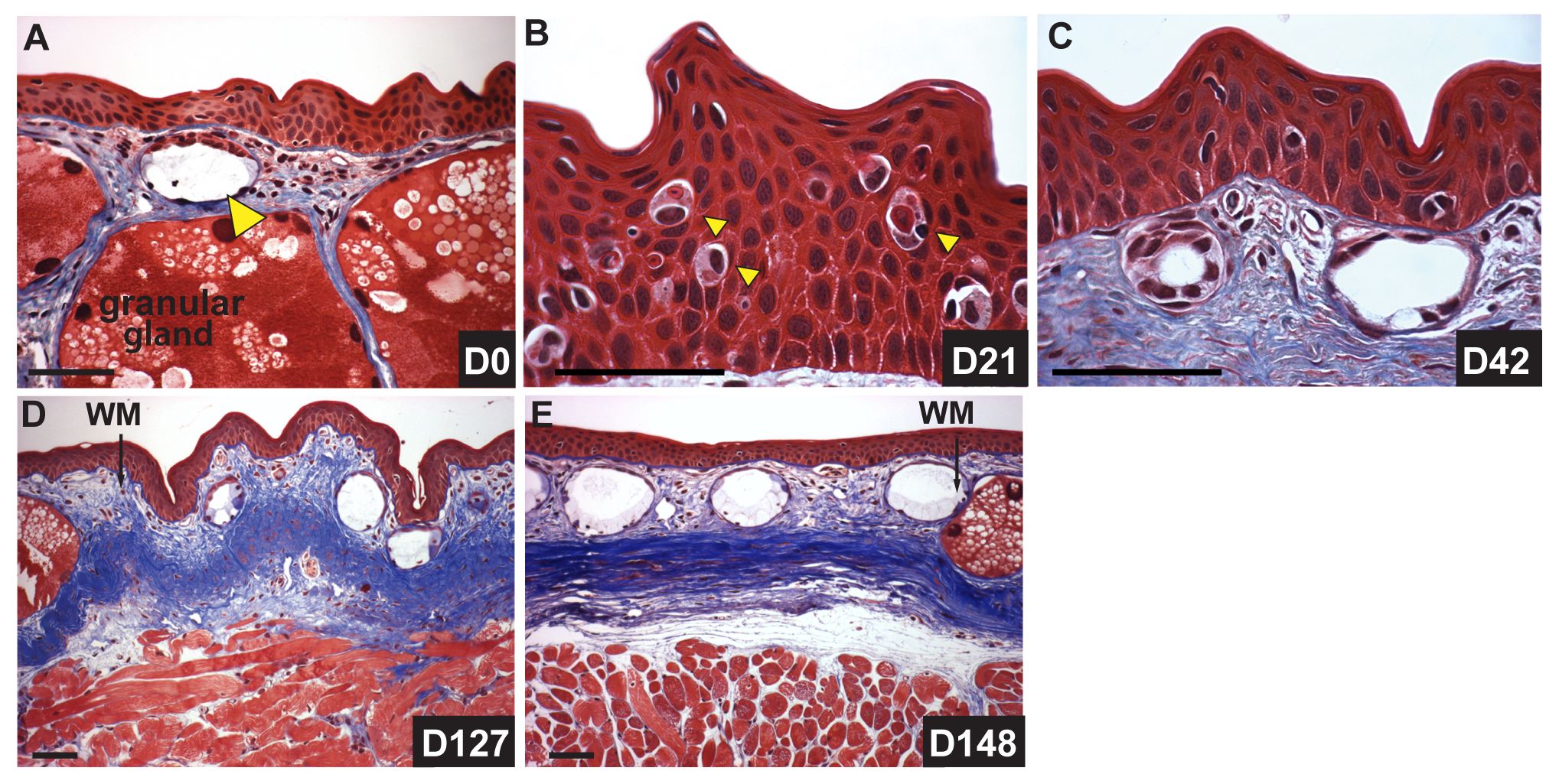

Supplement: Figure S3 — Detailed aspects of metamorphic axolotl skin regeneration over 147 days. A) Granular and mucous glands (yellow arrows) present in the stratum spongiosum. Collagen fibers are present between the glands. B) Some cellular aggregations in the epidermis appear to be early stages of regenerating glands (yellow arrows). C) Regenerated glands within densely compacted collagenous ECM beneath the epidermis. D) Stratum compactum is beginning to coalesce as the rest of the dermis has regenerated. Some fibrotic tissue remains within the regenerated muscle. E) Complete scar-free skin regeneration at D147 dpi. All tissue layers are present. Granular glands remain immature compared to uninjured skin. Scale bars = 100 µm. (TIF) [file pone.0032875.s003.tif]

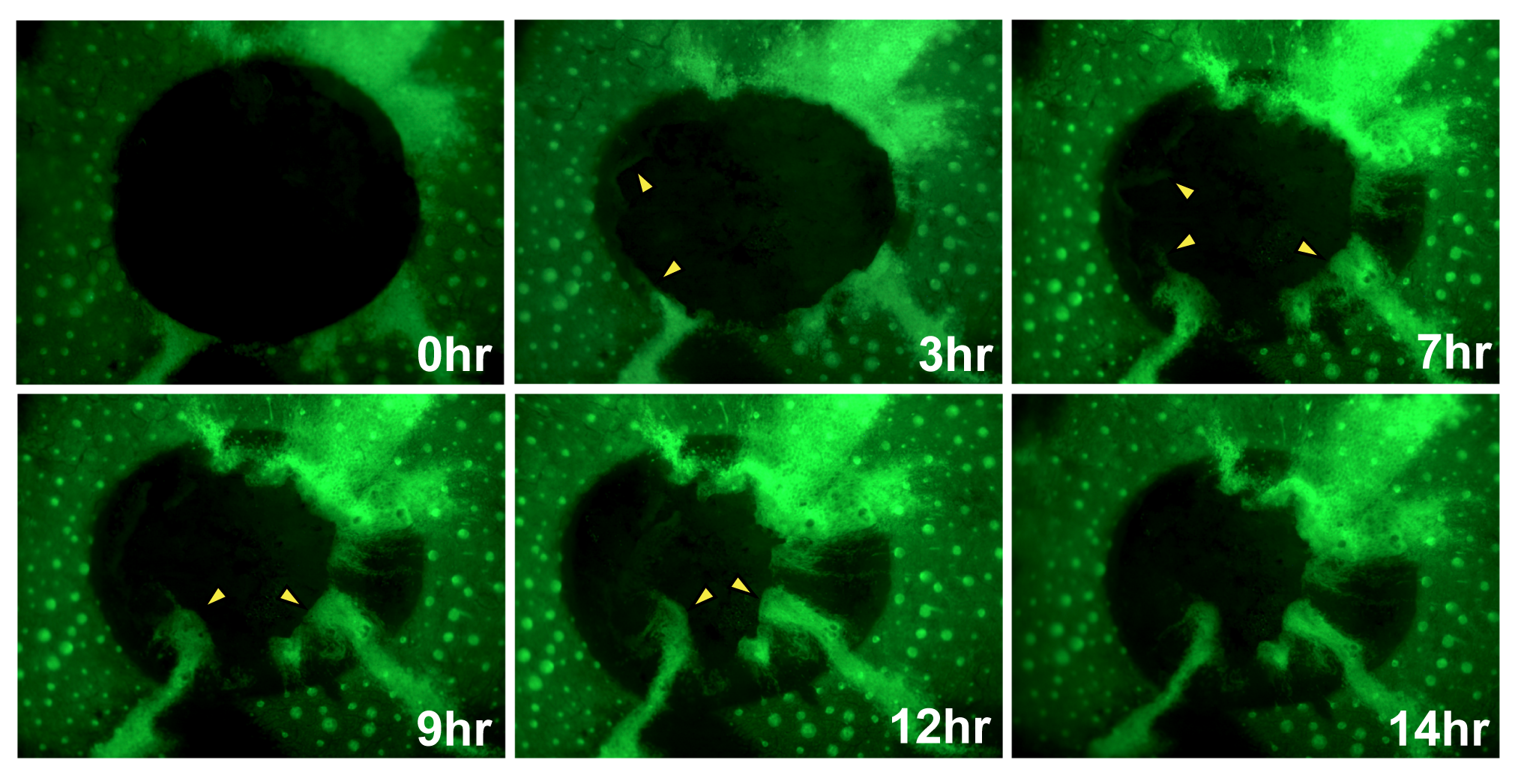

Supplement: Figure S4 — Visualizing the rate of re-epithelialization in paedomorphic axolotls using GFP transplanted skin. 1.5 cm×1.5 cm squares of dermis and epidermis from ubiquitously expressing GFP axolotls were transplanted to same size explanted areas on the tail of adult white axolotls. After transplants had healed 4mm FTE biopsy punches were made through the transplanted tissue and GFP-labeled epidermis was observed and photographed migrating to cover the wound bed over 18hrs. Migration was observed beginning 3hrs post injury and was complete between 18 and 24hrs. (TIF) [file pone.0032875.s004.tif]

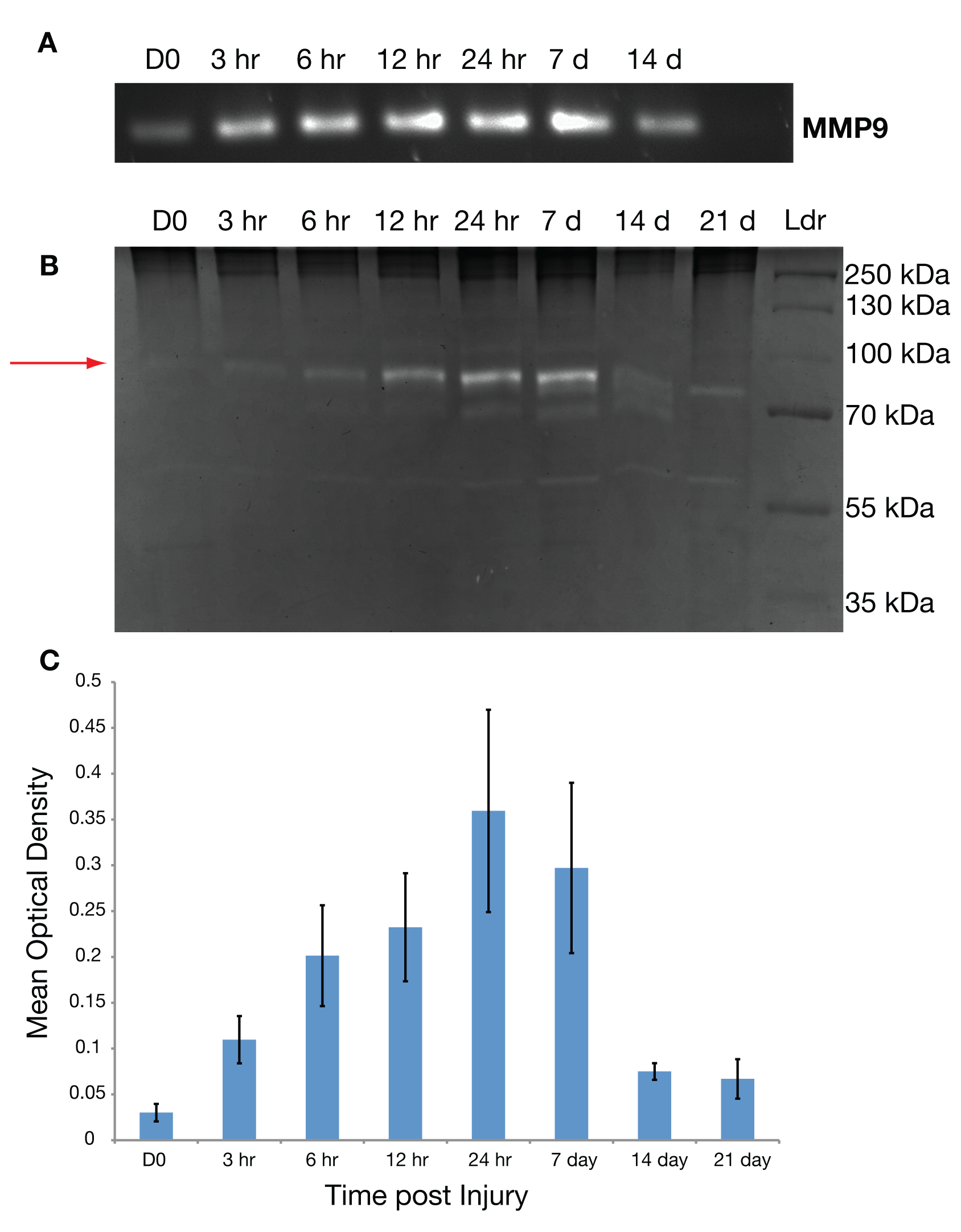

Supplement: Figure S5 — MMP transcription and activity are correlated during tail regeneration in paedomorphs. A) Axolotl MMP9 expression (semi-quantitative PCR) from tail tissue following amputation and through D14 post-amputation. Expression is downregulated 7 days post injury. B) Gelatin zymography was used to assess MMP activity over 21 days post injury. Red arrow points to tentatively assigned MMP9 position based on size (∼85 kDa) from previously published work in newt and axolotl (Vinarasky et al. 2005, Santosh et al. 2011). C) Gelatinase activity lags behind transcriptional upregulation and peaks at D7. For quantification of activity, gel images were inverted, mean optical density of individual black bands were calculated on an Gel Logic gel imaging system (Kodak) and light was blocked to calculate a baseline (black) reference for each measurement. Standard errors are reported. Regenerating tail tissue and tissue 0.5mm rostral to the amputation plane was collected at 0hrs, 3hrs, 6hrs, 12hrs, 24hrs, 7 d, 14 d, and 21 d after injury (n = 3 per time point), snap frozen on dry ice, and stored at –80°C. Tissues were homogenized for 10 minutes on ice in 1:4 (w:v) homogenization solution [50 mol/L Tris-Cl (pH 7.6), 150 mol/L NaCl, 1% (v/v) Triton-X100, 10 mol/L EDTA, 1 mol/L PMSF], sonicated, set on ice for 10 minutes, and spun at 14 000×g for 10 minutes at 4°C. Supernatants were decanted off, quantified using a BCA protein assay kit (Pierce), and stored at –80°C. 25 µg total protein was diluted in zymogram sample buffer (Bio-Rad) and electrophoresed on Ready Gel zymogram polyacrylimide gels containing gelatin (Bio-Rad). MMP proteins were re-natured by washing gels in zymogram renaturation buffer (Bio-Rad) for 30 minutes and incubated for 16 hrs at 37°C in development buffer (Bio-Rad). Gels were stained with 0.5% (w/v) Coomassie Blue R-250 (Bio-Rad) for 30 minutes and de-stained with an acetic acid, methanol, and dH20 solution (1∶5∶4) until clear bands were visible. (TIF) [file pone.0032875.s005.tif]

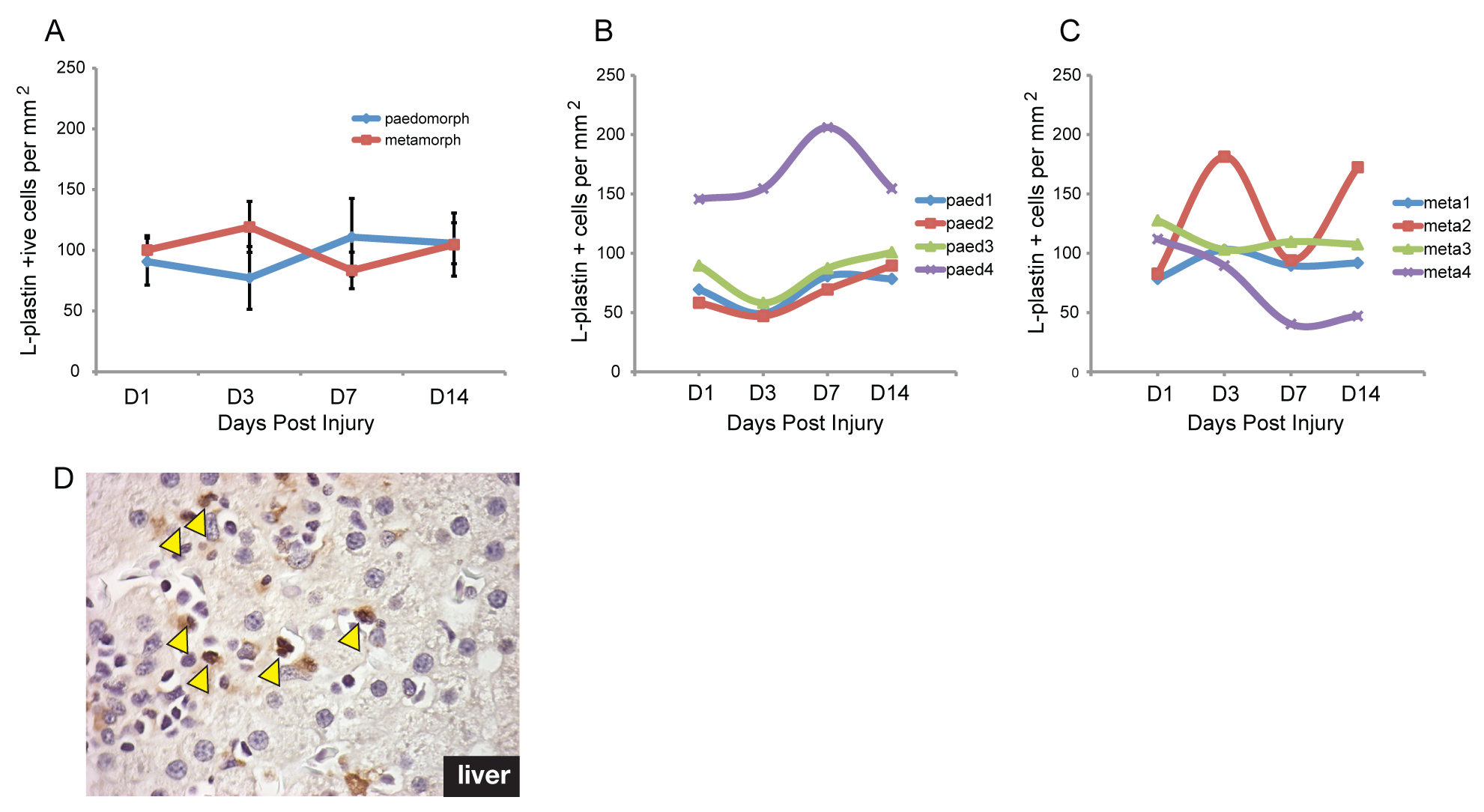

Supplement: Figure S6 — Individual variation in leukocyte numbers during scar-free healing. A) Total leukocyte numbers based on L-plastin staining for paedomorphs and metamorphs (n = 4 for each morph). Because multiple wounds were made on the same animals the inflammatory response could be tracked per individual. B) One paedomorph exhibited an unusually high inflammatory response (paed 4) and we removed it from our analysis. C) Variation across metamorphs revealed no outliers. D) Control staining for myloperoxidase in axolotl liver section. (TIF) [file pone.0032875.s006.tif]
