# Supplementary material for: Skin Regeneration in Adult Axolotls: A Blueprint for Scar-Free Healing in Vertebrates
Source: PLoS One. 2012 Apr 2;7(4):e32875. doi: 10.1371/journal.pone.0032875 (PMC3317654; doi:10.1371/journal.pone.0032875)
Supplement: Table S2 — Leukocyte profiles for Ambystoma mexicanum (paedomorph and metamorph). Numbers for each particular leukocyte type represented as a percentage of total leukocytes (thrombocytes were not counted). (DOC) [file pone.0032875.s008.doc]

**Table S2**. Leukocyte profiles for *Ambystoma mexicanum* (paedomorph and metamorph). Numbers indicate percent of total leukocytes (thrombocytes were not counted).

| Taxon | Species | Neutrophil | Eosinophil | Basophil | Lymphocyte | Monocyte |
| --- | --- | --- | --- | --- | --- | --- |
| Amphibians | *Ambystoma mexicanum*  (paedomorph) | 22.9 | 21.5 | 0.2 | 43.6 | 2.7 |
|  | *Ambystoma mexicanum*  (metamorph) | 18.4 | 12.2 | 0.9 | 55.8 | 5.0 |
